# Dynein Clusters into Lipid Microdomains on Phagosomes to Drive Rapid Transport toward Lysosomes

**DOI:** 10.1016/j.cell.2015.12.054

**Published:** 2016-02-11

**Authors:** Ashim Rai, Divya Pathak, Shreyasi Thakur, Shampa Singh, Alok Kumar Dubey, Roop Mallik

**Affiliations:** 1 Department of Biological Sciences, Tata Institute of Fundamental Research, Homi Bhabha Road, Mumbai 400005, India

## Abstract

Diverse cellular processes are driven by motor proteins that are recruited to and generate force on lipid membranes. Surprisingly little is known about how membranes control the force from motors and how this may impact specific cellular functions. Here, we show that dynein motors physically cluster into microdomains on the membrane of a phagosome as it matures inside cells. Such geometrical reorganization allows many dyneins within a cluster to generate cooperative force on a single microtubule. This results in rapid directed transport of the phagosome toward microtubule minus ends, likely promoting phagolysosome fusion and pathogen degradation. We show that lipophosphoglycan, the major molecule implicated in immune evasion of *Leishmania donovani*, inhibits phagosome motion by disrupting the clustering and therefore the cooperative force generation of dynein. These findings appear relevant to several pathogens that prevent phagosome-lysosome fusion by targeting lipid microdomains on phagosomes.

## Highlights

• Dynein clusters into lipid microdomains on the phagosome as it matures
• Clustering allows many dyneins to simultaneously contact a single microtubule
• Large cooperative forces can now be generated to transport phagosomes to lysosomes
• Leishmania lipophosphoglycans disrupt microdomains and inhibit this transport

## Graphical Abstract

**Figure:**
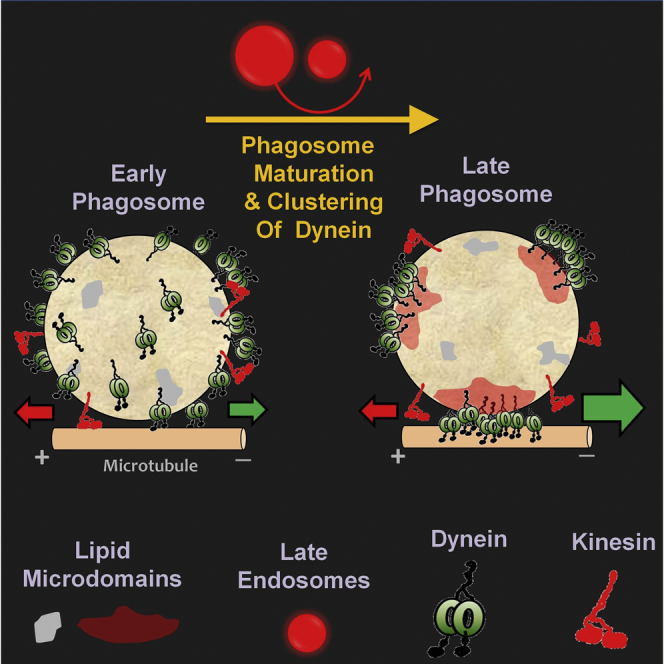

## Teaser

Dynein motors physically cluster into lipid rafts on the membrane of a phagosome as it matures inside the cell. The geometric organization in clusters increases the generation of force and allows many motors to cooperate to drive fast transport of the phagosome, a process required for elimination of many intracellular pathogens.

## Introduction

Microtubule motors of the kinesin and dynein families drive many cellular processes such as organelle transport, chromosome segregation, and beating of cilia/flagella. This diversity of function requires the cellular localization and activity of motors to be regulated in many ways (Vale, 2003). Regulation of motors at the single-molecule level by motor-associated regulatory proteins has been studied extensively (Vallee et al., 2012, Verhey and Hammond, 2009). However, most cellular functions require large forces that can only be generated collectively by a team of many motors (Mallik et al., 2013). Little is known about how such motor teams are assembled at appropriate cellular locations before they can execute a specific task. The substrate on which motor teams must assemble inside cells is usually a lipid membrane, for example, the bilayer membrane covering vesicular cargoes that are transported by motors. We therefore wondered if motor recruitment to a lipid membrane can be controlled by the membrane itself, perhaps in coordination with other membrane-bound proteins that regulate vesicle trafficking (e.g., Rab GTPases).

In this respect, the heterogeneity of biological membranes is of particular interest. Cholesterol and sphingolipids appear enriched within lipid microdomains (also known as lipid rafts), where they enhance membrane packing to promote microdomain formation (Mayor and Rao, 2004, Rao and Mayor, 2014, Simons and Ikonen, 1997). This process is likely facilitated by a combination of protein-lipid and protein-protein interactions, because microdomains are enriched in specific proteins (e.g., glycosylphosphatidylinositol [GPI]-anchored proteins) and may be maintained by active processes that drive the membrane away from thermodynamic equilibrium (Rao and Mayor, 2014). Motors could be localized to microdomains by direct binding to lipids (Klopfenstein et al., 2002) or via adaptor proteins (Johansson et al., 2007). Membranous regions of high motor density could potentially be created by clustering many copies of a motor within a microdomain. Such geometrical clustering may be of advantage if multiple motors are to work cooperatively as a team (Mallik et al., 2013, Rai et al., 2013). Geometrical arguments suggest that motor clustering is necessary for efficient transport of micron-sized cargoes (Erickson et al., 2011). Indeed, cooperative improvement in transport of artificial liposomes through clustering-induced dimerization of kinesin-3 motors has been reported (Klopfenstein et al., 2002). A minus-end-directed kinesin is also shown to localize into membrane domains near the apical subplasma membrane of polarized epithelial cells (Noda et al., 2001).

However, the functional relevance of clustering of motors and its impact on specific cellular processes is unknown. In this context, the appearance of microdomains on phagosomes with maturation is particularly interesting (Dermine et al., 2001, Dermine et al., 2005, Goyette et al., 2012). Phagocytosis and subsequent encapsulation of microbes into a membranous vesicle result in the formation of a phagosome. Phagosome maturation is intimately connected to microtubule (MT) motor-driven motion. Early phagosomes (EPs) move in a bidirectional (back-and-forth) manner on MTs, when they physically interact with and exchange lipids and proteins with endosomes (Blocker et al., 1997, Vieira et al., 2002). Intriguingly, this motion changes as the phagosome matures, so that late phagosomes (LPs) exhibit rapid unidirectional dynein-driven transport toward the MT minus end. The mechanism of this change is important to understand because it facilitates fusion of phagosomes with perinuclear lysosomes and is essential for pathogen clearance. MT depolymerization blocks delivery of fluid phase markers from endosomes to phagosomes and also reduces phagosome-lysosome fusion (Blocker et al., 1996, Desjardins et al., 1994, Harrison et al., 2003). Importantly, pathogens such as *Mycobacterium tuberculosis* (Sun et al., 2007) and *Salmonella* (Harrison et al., 2004) specifically inhibit this switch to dynein-dependent transport as a survival strategy.

We therefore wondered if microdomains on the phagosome membrane could upregulate dynein-driven transport of phagosomes. Cholesterol appears to be a major player in microdomain formation on cellular membranes (Mayor and Rao, 2004, Rao and Mayor, 2014, Simons and Ikonen, 1997). Dynein-driven transport of endosomes increases in cholesterol storage disorders like Niemann-Pick disease, where cholesterol-laden “paralyzed” endosomes cluster around the MT minus ends (Lebrand et al., 2002). Cholesterol accumulation into endolysosomes results in cholesterol-poor phagosomes that are unable to fuse with lysosomes (Huynh et al., 2008). Interestingly, the GTPase Rab7 that recruits dynein to phagosomes interacts with the cholesterol sensor ORP1L (Rocha et al., 2009) and is enriched in a cholesterol-rich detergent resistant fraction of phagosomal membranes (Goyette et al., 2012).

Taken together, the above observations suggest a molecular connection between dynein, Rab7, and cholesterol within microdomains on the phagosome membrane. Here, we show using multiple experimental approaches that dynein clusters into microdomains on the membrane of a phagosome as it matures inside cells. This geometrical clustering allows many dyneins to simultaneously contact a single MT and generate large cooperative force. This force drives rapid retrograde transport of late phagosomes (LPs), likely enabling their fusion with degradative lysosomes. We also show that lipophosphoglycan, the main molecule used by pathogenic *Leishmania donovani* parasites to survive inside macrophages, specifically disrupts the clustering of dynein on LP membranes to block retrograde transport of LPs.

## Results

### Transport- and Maturation-Dependent Changes of Latex Bead Phagosomes Inside Cells

We used phagocytosed beads (latex or silica) to understand how motor-driven transport and phagosome maturation impact each other. Phagocytosed beads undergo biogenesis inside cells to acquire a bilayer lipid membrane (Desjardins and Griffiths, 2003). Motor and non-motor proteins assemble in situ on this membrane to drive vigorous transport of these “latex bead phagosomes” (henceforth referred to as phagosomes) along MTs (Blocker et al., 1997, Rai et al., 2013). Motion at defined stages of maturation can be assayed using a pulse-chase strategy that allows phagosomes to mature for specific chase periods after ingestion. Proteomic and biochemical studies have extensively used latex bead phagosomes to understand phagosome biology (Desjardins and Griffiths, 2003, Desjardins et al., 1994). These refractile and spherical phagosomes are also ideal for optical trapping to measure forces generated by motors (Rai et al., 2013).

**Figure 1:**
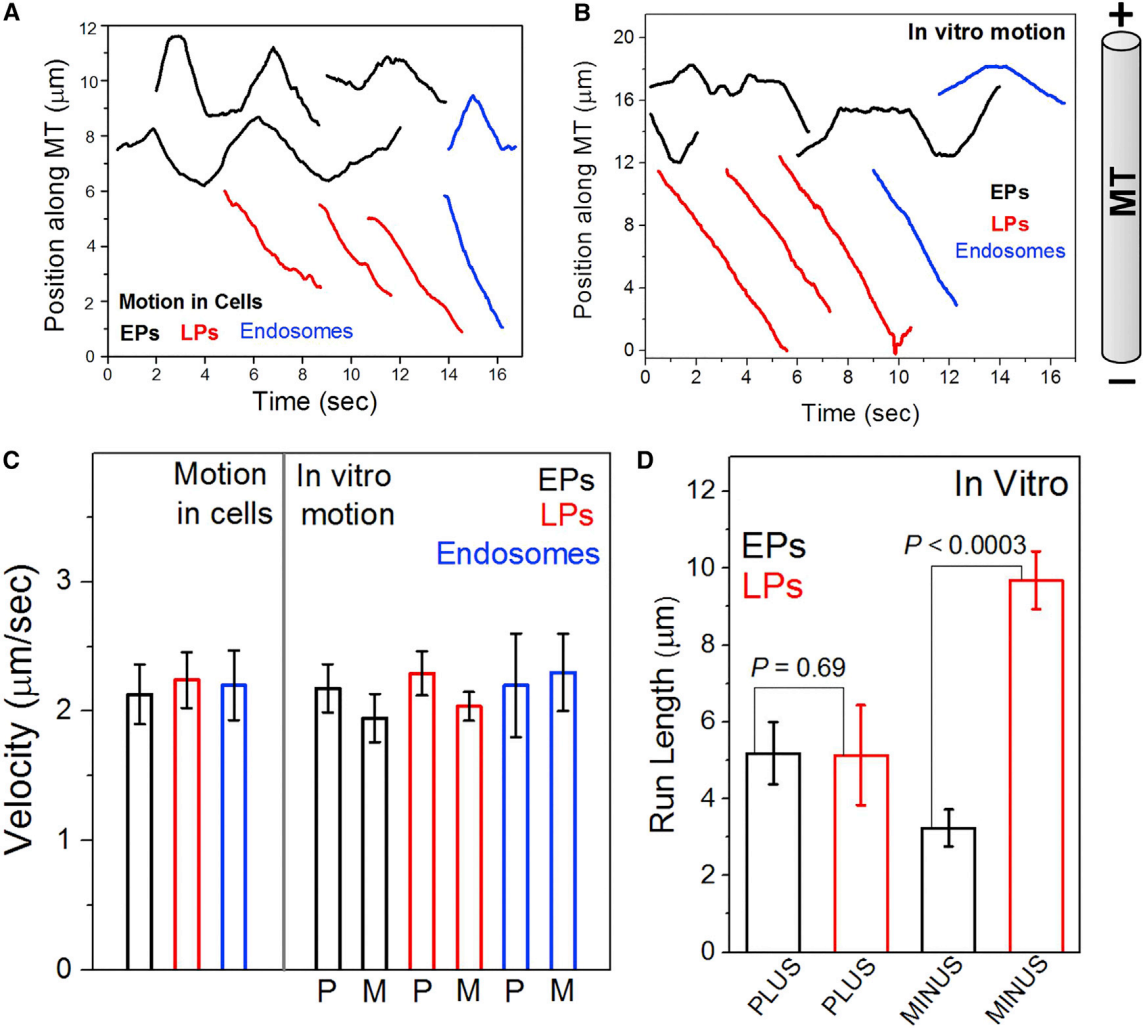
Latex Bead Phagosome Motion Inside *Dictyostelium* Cells and in Cell Extract (A) Position-time plots of early phagosomes (EPs), late phagosomes (LPs), and endosomes inside *Dictyostelium* cells. The distance traveled along a curved trajectory (assumed to be a microtubule) was calculated as a function of time. MT orientation inside cells is uncertain. (B) Position-time plots of purified EPs, LPs, and endosomes moving along single microtubules in an in vitro motility assay. The microtubule orientation is shown on the right. (C) Velocity of motile EPs, LPs, and endosomes inside *Dictyostelium* cells and in vitro. Direction of motion (toward plus or minus end of MT) is uncertain inside *Dictyostelium* cells and has therefore not been assigned. Plus (P)- or minus (M)-directed motion is indicated for in vitro motion. Error bars show SEM. One-way ANOVA shows no statistical difference between these velocities. (D) In vitro run length of EPs and LPs (35 of each analyzed). Error bars represent SEM. See also Figures S1 and S2.

**Figure S1:**
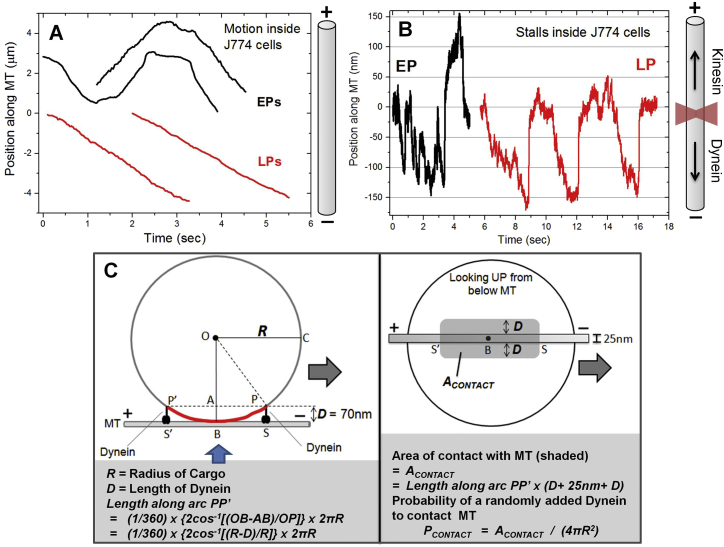
Motion and Force Generation of Phagosomes Inside Macrophage Cells: Calculation of the Area *A*_*CONTACT*_ from Where Dyneins Can Contact an MT to Drive Cargo Transport, Related to Figures 1 and 3 (A) Representative video tracks of early phagosomes (EPs) and late phagosomes (LPs) moving inside J774 mouse macrophage cells. MT orientation is shown as a cartoon. Approximately linear (X-Y) trajectories were chosen, and the component of motion along a straight line (assumed to be a single MT) was calculated. EPs usually exhibit bidirectional motion, but LPs moved in unidirectional manner along the MT. (B) Stall force records of EPs and LPs inside J774 cells are shown. The microtubule orientation (inferred from morphology of cells; see Rai et al., 2013) is also schematized along with an optical trap (red focused beam). EPs exhibit bidirectional stalls, but LPs show unidirectional stalls largely in the minus direction. This is broadly consistent with the stall records seen on EPs and LPs purified from *Dictyostelium* (main text). (C) Left: A spherical cargo of radius *R* is shown in contact with a MT at the bottom of the cargo (contact point = B). Two dynein molecules (each of length *D =* 70nm) are attached to points P and P’ on the cargo, and at S and S’ on the MT. P’S’ = PS = *D*. The permissible arc along which cargo-bound dyneins can contact the MT is PP’ (shown in red). Dyneins attached to cargo beyond this arc are not long enough to reach the MT. The projection of arc PP’ on the MT is SS’, and is the maximum length along this MT on which cargo-bound dyneins can engage. The direction of dynein driven motion is shown (arrow). Right: The same cargo is now visualized looking upward from beneath the MT (see blue arrow in left panel). Dyneins situated exactly atop the MT (along SS’) can engage the MT. Dyneins that are attached to cargo within a distance *D* perpendicular to the MT may also be able to reach the MT. Therefore, the maximum possible area of contact for dyneins ( = *A*_*CONTACT*_) can be approximated as the shaded rectangle (though this is likely an overestimate of the contact area). Bottom (gray box) Arc length PP’ and *A*_*CONTACT*_ are calculated in terms of *R* and *D*. If dyneins are randomly placed on the cargo, then the probability of dynein to attach within *A*_*CONTACT*_ is the ratio of *A*_*CONTACT*_ to total surface area of cargo ( = 4*πR*^2^). This probability is called *P*_*CONTACT*_, and is plotted as a function of *R* in Figure 3E (main manuscript).

Beads were phagocytosed into J774 mouse macrophages (Rai et al., 2013) or into *Dictyostelium discoideum* cells. The beads were chased inside *Dictyostelium* cells for 5–10 min to investigate early phagosome (EP) motion and for >30 min to investigate LP motion (Barak et al., 2014). EPs moved in bidirectional manner inside agar-flattened *Dictyostelium* cells (Figure 1A; Movie S1). For EPs, fast unidirectional segments of motion were interrupted by pauses followed by reversal and rapid unidirectional motion in opposite direction. In contrast, LP motion was largely uninterrupted and unidirectional (Figure 1A). Many LPs appeared to move smoothly inside cells over distances longer than apparent from Figure 1A, but the convoluted trajectories prevented reliable characterization of motion. Figure 1A also shows representative tracks of endosomes inside *Dictyostelium* cells. These endosomes do not have beads inside them but are highly motile endogenous vesicles at various stages of maturation. The motion of EPs was very similar to the bidirectional motion of endosomes, but the motion of LPs resembled that of unidirectional endosomes. The velocity of motile EPs within fast unidirectional segments was similar to unidirectional velocity of LPs and endosomes (Figure 1C). The bidirectional (EP) and unidirectional (LP) motions are also reproduced for phagosomes inside J774 mouse macrophages (Figure S1A). Therefore, phagocytosed beads appear to replicate specific maturation-dependent aspects of motion within the endophagosomal pathway. Phagosomes mature beyond the EP stage within ∼10 min of ingestion in *Dictyostelium* (Barak et al., 2014). It was therefore practically impossible to assay inside *Dictyostelium* cells how motor function changes from EPs to LPs.

### In Vitro Reconstitution of Early and Late Phagosome Transport

**Figure S2:**
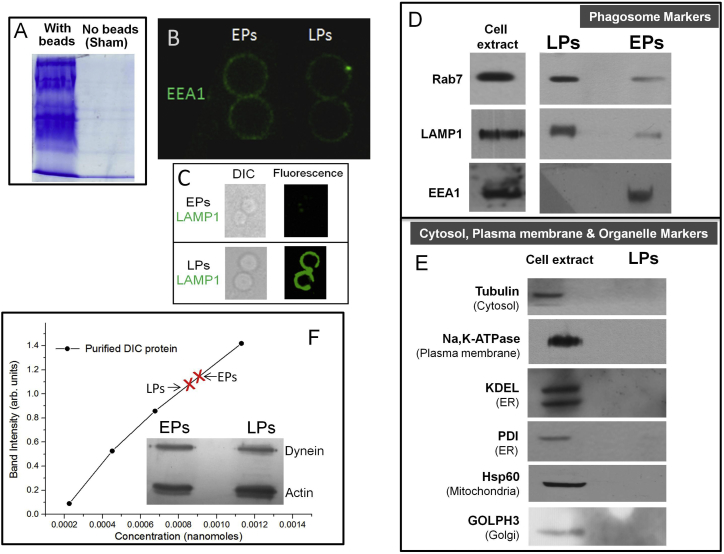
Identity and Purity of Latex bead Phagosomes and Western Blotting to Investigate Differences in Dynein Amount on Purified EPs and LPs, Related to Figures 1 and 3 (A) SDS PAGE of the 10%–25% interface of sucrose gradient after ultracentrifugation in a typical phagosome preparation. Results are shown for a latex bead phagosome prep (*Dictyostelium* cells + phagocytosed beads) and a sham prep (Equal number of *Dictyostelium* cells, but no beads added). No proteins are detected at the 10%–25% interface in the sham prep. Similar results were obtained with silica bead phagosomes. (B) Immuno fluorescence staining of EPs and LPs purified from J774 cells against EEA1 under identical imaging conditions. Two EPs and two LPs are shown. Note the stronger staining of EEA1 on EPs. (C) Fluorescent staining (right panels) of purified phagosomes using antibody against LAMP1 (late phagosome marker) shows enrichment of LAMP1 on purified LPs, but not on EPs. DIC images are also shown (left panels). (D) The identity of latex bead phagosomes is examined using two markers for late phagosomes (Rab7, LAMP1) and one marker for early phagosomes (EEA1). Rab7 and LAMP1 are enriched on purified LPs, but present in significantly smaller quantity in the EP sample. EEA1 (early endosome antigen 1) can be detected on purified EPs, but not in the LP sample. (E) The purity of latex bead phagosomes is examined using markers against Cytosol (tubulin), Plasma membrane marker (Na,K-ATPase), Endoplasmic Reticulum (KDEL and PDI), Mitochondrial marker (Hsp60), and Golgi marker (GOLPH3). Marker proteins against cytosol and other organelles can be detected in the cell lysate, but are not detected in the phagosome fraction. This suggests that the latex bead phagosome fraction is largely free of membrane and cytoplasmic contamination. For further details, see Section 6 of Supplemental experimental Procedures (under subheading “Purity”). (F) Inset shows western blots for dynein and actin (loading control) using early phagosomes (EPs) and late phagosomes (LPs) purified from J774 cells. An antibody against dynein intermediate chain (DIC) was used to detect dynein. Quantitative immunoblotting for DIC protein:- full length rat DIC protein with a His_6_ tag was expressed in bacteria and purified using Ni^+2^-NTA affinity. A dilution series of recombinant DIC was prepared and subjected to western blot experiment. Band intensity in western blot varied almost linearly with dilution. The EP and LP samples fall within this range of intensities (average intensity values of EPs and LPs indicated with red “x” mark). This experiment suggests that the amount of dynein on EPs and LPs is approximately equal. This experiment was repeated on four EP and four LP preparations. Phagosome-associated actin on the purified EPs/LPs was used as a loading control. The ratio of intensities for dynein band (EP:LP) was obtained after correcting for differences in actin intensity (i.e., differences in loading). This ratio was close to unity ( = 0.9 ± 0.3; mean ± s.e.m; 4 experiments), suggesting no additional recruitment of dynein on LPs. In our experience with in vitro motility of dynein-coated beads, many fold higher dynein is required to transition from single-dynein driven runs to runs of ∼6μm or longer. Small differences in amount of dynein below the threshold of our detection are therefore unlikely to bring about the significantly improved retrograde transport and very long runs of LPs (> 10μm, see Figure 1B).

Vesicle transport can be reconstituted in *Dictyostelium* cell extract, permitting controlled evaluation of motor protein activity (Pollock et al., 1999, Soppina et al., 2009b). Detailed protocols have been described (also see Experimental Procedures) for purification of EPs and LPs from *Dictyostelium* using a pulse-chase strategy (Barak et al., 2014, Gotthardt et al., 2006). Phagosomes purified from *Dictyostelium* were used for in vitro motility assays (Figure 1B; Movies S2 and S3) on polarity-labeled MTs (Soppina et al., 2009a). We verified the identity and purity of EPs and LPs purified in a similar manner from J774 or RAW264.7 mouse macrophage cell lines (Experimental Procedures; also see Figure S2). A clear difference was observed (Figure 1B) between motion of purified EPs (bidirectional with frequent reversals) and LPs (unidirectional retrograde, with rare reversals). In vitro motion of EPs and LPs was characteristically similar to their corresponding motion inside cells (compare Figures 1A and 1B). Figure 1B also shows bidirectional and unidirectional tracks of endosomes purified from *Dictyostelium*, which appear very similar to EPs (bidirectional) and LPs (unidirectional). The velocity of purified EPs, LPs, and endosomes during uninterrupted segments of motion was statistically same as their respective velocities inside cells (Figure 1C). Figure 1D reports run lengths for purified EPs and LPs (35 of each used for analysis). A run was defined as a period of uninterrupted fast motion (Rai et al., 2013, Soppina et al., 2009b). No difference in plus-directed run lengths was seen between EPs and LPs. In contrast, minus-end-directed LPs usually moved much farther than EPs (Figure 1D). The actual run length is likely larger for these LPs, because they often got stuck at obstacles or reached the end of the MT during in vitro motion. Note that runs of EPs could end in reversals or detachments from the MT. The persistent long minus-end-directed runs of purified LPs along single MTs is reminiscent of similar observations inside mouse macrophages (Rai et al., 2013) and may facilitate degradative fusion of phagosomes with lysosomes (Blocker et al., 1997, Harrison et al., 2003).

### Force Measurement on Early and Late Phagosomes

**Figure 2:**
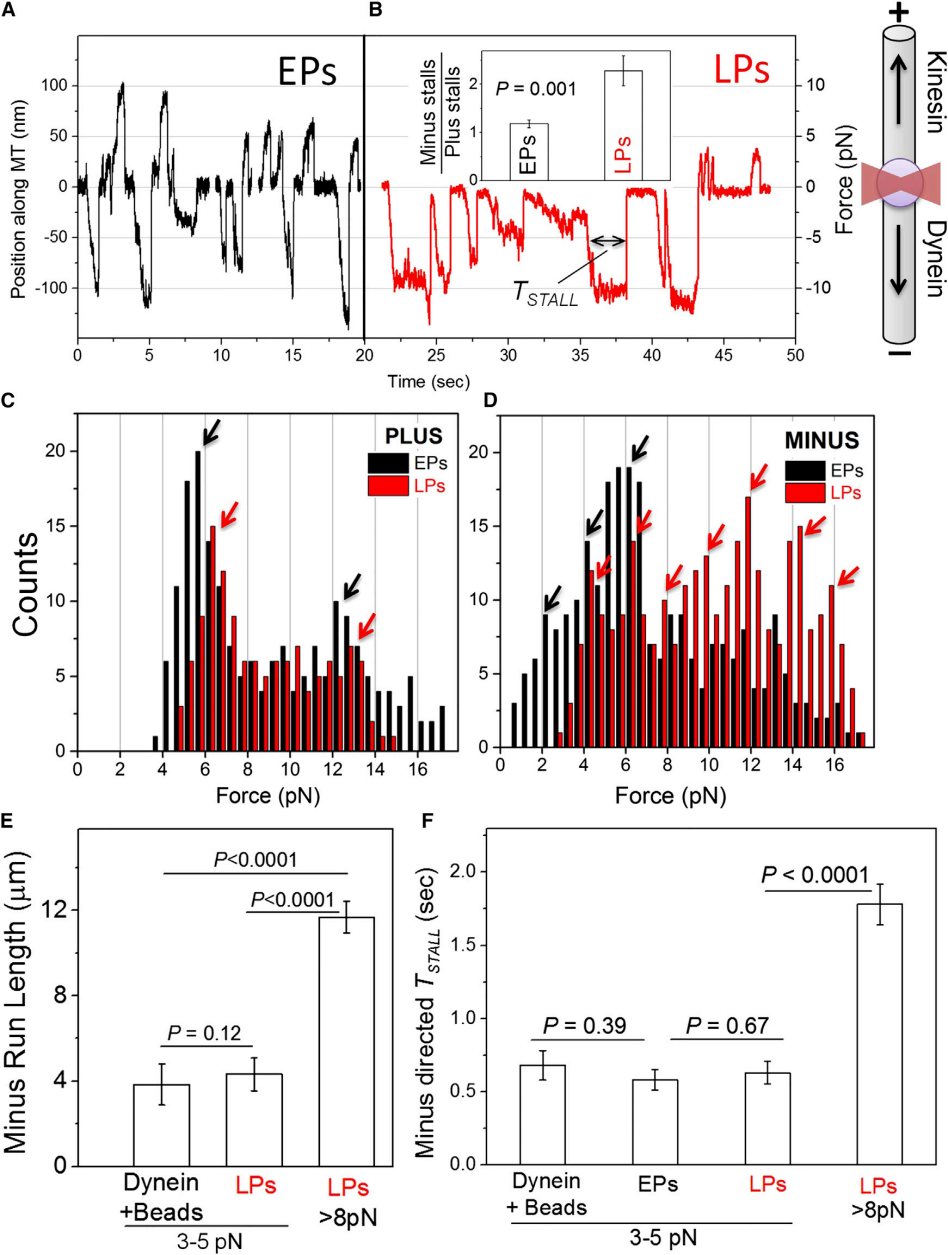
Optical Trapping of EPs and LPs Reveals Differences in Dynein Number, Function, and Cooperativity (A) Stall force records of three individual EPs purified from *Dictyostelium*. The corresponding force is also shown. A schematic on the right shows the microtubule orientation, a trapped phagosome (purple sphere), and focused optical trap beam (red). These are not drawn to scale. The direction of force generation by kinesin and dynein is also shown. (B) Stall force records for a single LP purified from *Dictyostelium*. Inset shows the ratio of minus:plus stalls on EPs and LPs. EPs show equal number of stalls in both directions, but LPs show twice as many minus stalls. Error bars represent SEM. (C) Stall force histogram for plus-directed stalls of EPs and LPs purified from *Dictyostelium*. Both histograms are similar with peaks at ∼6 pN and ∼12 pN (arrows). (D) Stall force histogram for minus-end-directed (dynein driven) stalls of EPs and LPs purified from *Dictyostelium*. A clear shift toward higher forces is seen in the LP data, suggesting more active dyneins on LPs. Arrows with ∼2 pN periodicity indicate peak positions (see text). (E) Minus-end-directed run lengths for beads coated with purified dynein and LPs, both generating force between 3 and 5 pN. Run length for LPs generating force >8 pN is also shown. Minimum of 19 runs analyzed for each condition. Error bars show SEM. (F) Time for which dyneins survive against half-maximal load (*T*_*STALL*_; see Figure 2B) is plotted for dynein-coated beads, EPs, and LPs. The *T*_*STALL*_ is similar for beads, EPs, and LPs at low force (between 3 and 5 pN). *T*_*STALL*_ is higher for LPs generating >8 pN force. Minimum 20 stalls used for each condition. Error bars represent SEM. See also Figure S1.

The above data show that a switch from bidirectional (EP) to retrograde unidirectional (LP) motion in the phagosomal/endosomal pathway is reproduced in our in vitro motility assays. To understand the mechanism of this switch, we measured the force generated by motors on purified EPs and LPs using an optical trap (Experimental Procedures). Motors on EPs generated vigorous force in both directions, with frequent transitions between plus and minus-end-directed stalls (Figure 2A). In contrast, LPs exhibited repeated minus-end directed stalls (Figure 2B). The total number of plus and minus stalls were counted (23 EPs and 21 LPs used) to determine the ratio of minus:plus stalls (Figure 2B, inset). The significant increase in this ratio for LPs showed that dynein driven stalls dominate as the phagosome matures and likely cause the minus-end-directed bias in LP motion. Qualitatively similar differences in force generation between EPs (bidirectional) and LPs (minus-end directed) were also seen for phagosomes inside J774 cells (Figure S1B).

Figure 2C shows a histogram for plus-directed (kinesin-driven) stall forces on EPs and LPs. The histogram for EPs and LPs appears similar, with a major peak at ∼6 pN followed by a broad distribution centered at ∼12 pN. This is similar to observation on LPs inside J774 cells (Rai et al., 2013). We have earlier shown using purified kinesin-coated beads that the *Dictyostelium* Unc104 kinesin generates ∼6 pN force (Soppina et al., 2009b). Therefore, one or two kinesins appear to drive the motion of EPs as well as LPs, in agreement with the similar plus-directed run length of EPs and LPs (Figure 1D). It therefore appears that kinesin activity on phagosomes is not sensitive to maturation. Enhanced retrograde transport of LPs therefore does not arise from a suppressed kinesin activity on LPs. In contrast to the observation for kinesin, minus-end-directed stalls showed a pronounced shift toward higher force on LPs (Figure 2D). A cumulative frequency count showed that only 50% EPs exerted >6 pN force, but this fraction was 86% for LPs. The major peaks for minus-end-directed LPs appeared at ∼4, 6, 8, 10, 12, 14, and 16 pN (Figure 2D, red arrows), whereas the peaks for EPs were at ∼2, 4, and 6 pN (Figure 2D, black arrows). We have earlier shown using purified dynein-coated beads that single *Dictyostelium* dynein generates ∼1.1 pN force, similar to mammalian dynein (Soppina et al., 2009b). The ∼2pN interval between peaks in the force histogram agrees with our earlier observation on LPs inside J774 cells (Rai et al., 2013). This possibly happens because dynein is recruited in pairs to LPs via a dimer of Rab7, with each dynein-pair generating 1.1 × 2 ∼2 pN force (Rai et al., 2013). Dynein is recruited to early endosomes/phagosomes by Rab5, which is also a dimer in the active GTP-bound conformation (Daitoku et al., 2001).

Optical trapping therefore confirmed an increase in frequency and magnitude of minus-end-directed force generating events on LPs. Since force measurements suggested similar activity of one or two kinesins on EPs and LPs (Figure 2C), the improved minus-end-directed motion must stem from enhanced dynein activity on LPs. Such enhancement could arise in one or more of the following ways: (1) molecular properties and function of dynein is different on LPs compared to EPs; (2) there is more dynein on LPs than on EPs; or (3) organization of dynein on the LP membrane is different from the EPs, allowing more dyneins to generate force simultaneously. In what follows, we will examine the evidence pertaining to each of these possibilities.

### No Significant Difference in Molecular Function and Amount of Dynein between Early and Late Phagosomes

As already mentioned, minus-end-directed velocities for EPs and LPs were statistically the same (Figure 1C). Both EPs and LPs showed a 2 pN periodicity in force histograms (Figure 2D), suggesting that the force generated by a pair of dyneins is similar. Therefore, each dynein on EPs as well as LPs likely generates ∼1 pN force, which is the same as the force generated by purified *Dictyostelium* dynein-coated beads (Soppina et al., 2009b). We next measured minus-end-directed run lengths of dynein-coated beads and LPs (both 759 nm diameter) that were first made to stall against the optical trap before release and free motion on the MT (Mallik et al., 2005). We chose only LPs that generated 3–5 pN force to enable a fair comparison with dynein-coated beads. The average stall force was statistically same for beads and such LPs (3.13 ± 0.66 pN and 3.29 ± 0.56 pN, respectively; p = 0.3), confirming motion driven by approximately the same number of dyneins. The run length was also statistically same for dynein-coated beads and LPs generating 4–6 pN force (Figure 2E). This suggests no significant increase in dynein’s processivity through association with LP-specific regulatory proteins. As expected, LPs exhibiting higher force in minus direction had longer runs because they were likely driven by more dyneins (Figure 2E).

Next, we estimated the persistence of dyneins against load by measuring the time spent by cargo above half-maximal load against an optical trap (*T*_*STALL*_; see double-headed arrow in Figure 2B). Again, stalls within the same force regime showed statistically same *T*_*STALL*_ for beads, EPs, and LPs (Figure 2F). Taken together, velocity, force, run length, and *T*_*STALL*_ suggest no significant change in the molecular function of dynein with phagosome maturation. The higher net-minus force on LPs therefore likely results from a larger number of dyneins that can engage a MT simultaneously to drive LP motion. This is supported by additional peaks of ∼2 pN periodicity at higher force for LPs (Figure 2D).

**Figure 3:**
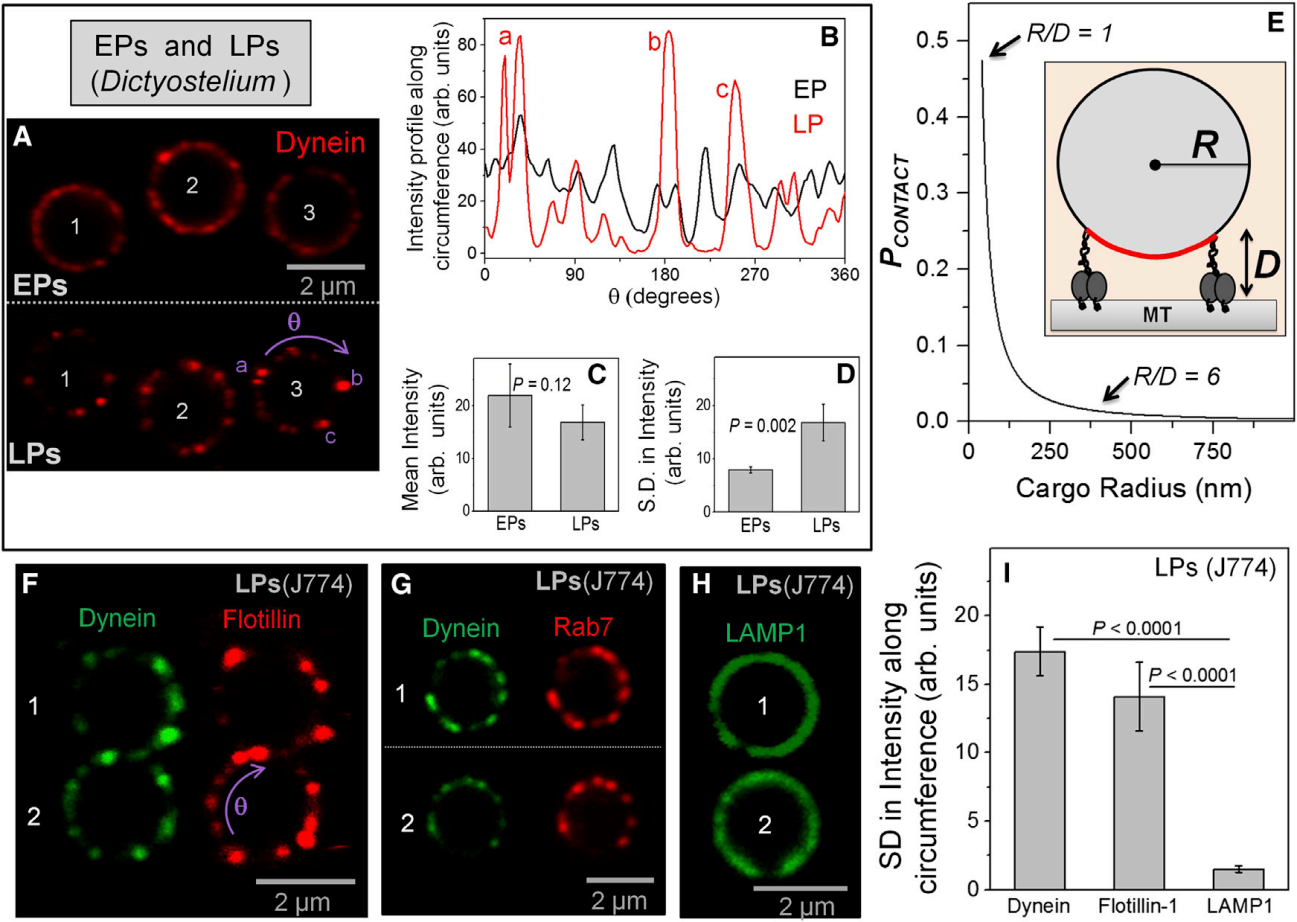
Clustering of Dynein, Rab7, and Flotillin into Cholesterol-Rich Domains on Late Phagosomes (A) Confocal images showing dynein immunofluorescence staining for three EPs (top; numbered 1, 2, and 3) and three LPs (bottom; numbered 1, 2, and 3). The EPs/LPs were purified from *Dictyostelium*. Dynein is detected using an antibody raised against *Dictyostelium* dynein heavy chain. The phagosomes are 2 μm in diameter. Note the comparatively uniform staining of dynein along circumference of EPs but distinctly punctate staining along LPs. Three puncta are indicated (as a, b, and c) on LP#3. Angular position along phagosome circumference is measured using the rotation angle (θ). (B) The pixel intensity for dynein staining along circumference of EP#3 and LP#3 (see Figure 3A) is plotted as a function of angle (θ). The peak positions (a, b, and c) correspond to the puncta on LP#3 in Figure 3A. (C) The mean pixel intensity (calculated along eight EPs and seven LPs) is statistically the same for EPs and LPs, suggesting that no significant change in dynein as a function of phagosome maturation. Error bars represent SD. (D) Fluctuation in dynein staining intensity along phagosome membrane is estimated from the SD in intensity measured along the circumference. EPs have lower fluctuation (suggesting uniform staining) compared to LPs (punctate staining). Error bars represent SD. (E) Probability (*P*_*CONTACT*_) that a dynein motor of size *D* added in uniform (nonclustered) manner onto a spherical cargo of radius (*R*) will be able to contact a MT at the bottom of the cargo. This suggests that it is very difficult for many (more than five) randomly distributed dyneins to simultaneously engage a MT to transport an LP (see text). Inset shows a spherical cargo of radius *R* with two dyneins (each of length *D*) attached to it. The dyneins heads are also in contact with a microtubule (MT) at the base of the cargo. The maximum arc of contact for dyneins along the cargo surface is shown (red). (F) Confocal double immunofluorescence staining of two individual LPs (numbered 1 and 2) purified from J774 cells for dynein (green) and flotillin-1 (red). Dynein is detected using an antibody against dynein intermediate chain. The LPs are 2 μm in diameter. Dynein and flotillin show a very similar punctate pattern on each LP and therefore appear to colocalize. (G) Confocal double immunofluorescence staining of two LPs (numbered 1 and 2) purified from J774 cells for dynein (green) and Rab7 (red). Dynein and Rab7 show similar punctate patterns, suggesting colocalization into puncta. (H) Confocal immunofluorescence staining of LAMP1 (LP marker) on two LPs (numbered 1 and 2) shows a continuous and uniform distribution of LAMP1. (I) The SD of fluorescence intensity along LP circumference is plotted for dynein, flotillin-1, and LAMP1 (five LPs used for each). Error bars represent SD. See also Figures S1, S2, S3, and S4.

We next probed the obvious possibility that improved retrograde motion is caused by increased recruitment of dynein on LPs. Immuno-electron microscopy of phagocytosed beads in J774 cells has reported no increase in dynein on LPs compared to EPs, and no increase was seen on late endosomes compared to early endosomes (Habermann et al., 2001). Figure 3A shows three representative EPs and three LPs (numbered in the figure) that were purified from *Dictyostelium*. Dynein consistently showed a non-uniform punctate distribution on LPs, but not so on EPs. We traced a circle along the circumference of individual EPs/LPs (Experimental Procedures) to determine the fluorescence intensity as a function of the angular rotation (θ; shown in Figure 3A). Figure 3B shows representative profiles of EP#3 and LP#3 (see numbering in Figure 3A). The EP shows high basal values of pixel intensity and smaller fluctuations. In contrast, the baseline for LP intensity is almost zero, but there are strong peaks corresponding to intense puncta (puncta on LP#3 and corresponding peaks in Figure 3B are marked as a, b, and c). The mean pixel intensity after averaging along the circumference for eight EPs and seven LPs is statistically the same (Figure 3C). This suggests no significant difference in dynein amount between EPs and LPs at the single-phagosome level. Quantitative western blotting confirms that the amount of dynein does not change significantly between purified EPs and LPs (Figure S2F). Figure 3D plots the SD in pixel intensity along the circumference for EPs and LPs. The SD is significantly higher for LPs, suggesting that dynein intensity is nonuniform on LPs. These data suggest that dynein redistributes from more uniform organization (on EPs) to a punctate organization on LPs, where it presumably clusters within small domains. This clustering appears to occur with no significant recruitment of additional dynein on LPs.

### Clustering of Dynein and Why This Is Needed for Transport of Large Cargoes

The punctate staining provided preliminary evidence that dynein exists in clusters on the LP membrane (Figure 3A). We observed approximately eight puncta of dynein (see LPs in Figure 3A) along an LP of 2 μm diameter (circumference = 6.28μm). The number of puncta per unit length (and per unit area) was calculated, yielding ∼20 puncta on the entire surface of such LPs. Assuming that the inter-puncta distance is maintained, the approximately 6-fold smaller surface area of a 759-nm-diameter LP (used for motility) yields only approximately three puncta on its entire surface. The long minus runs (Figures 1B and 1D) and high force (up to ∼16 pN; Figure 2D) therefore suggest that the motile LPs are largely driven by multiple dyneins within a single puncta.

A spherical cargo of radius *R* is schematized in Figure 3E (inset) with two dyneins (each of length *D*) attached to the cargo and also engaged to a MT. If their attachment points on the cargo are moved further upward, then the dyneins cannot reach the MT. The maximum arc along which dyneins can contact the MT is shown in red. Dyneins situated along this arc within distance ∼*D* perpendicular to the plane of paper may also reach the MT (Figure S1C). Therefore, there exists an approximately rectangular contact area on the cargo ( = *A*_*CONTACT*_), such that only dyneins within *A*_*CONTACT*_ can drive transport along a single MT. An expression for *A*_*CONTACT*_ is derived in Figure S1C. As an example, *A*_*CONTACT*_ ∼0.09 μm^2^ for a spherical cargo of 1 μm diameter with surface area of 3.14 μm^2^. If we now randomly place motors on the spherical cargo, the probability that a motor will fall within *A*_*CONTACT*_, and therefore engage the MT, is *P*_*CONTACT*_ *= A*_*CONTACT*_ */* (*4πR*^*2*^). Here, the denominator is the total surface area of the spherical cargo. We plot *P*_*CONTACT*_ as a function of cargo radius in Figure 3E, with *D* = 70 nm. For neuronal vesicles with a diameter of ∼100 nm (Hendricks et al., 2010), there is an ∼50% probability that an added dynein will be able to drive transport. However, *P*_*CONTACT*_ reduces rapidly with increasing cargo size. For an EP or LP used in our motility assays (*R* = 380 nm and *R/D* ∼6), a randomly added dynein would have only a 4% chance of contacting the MT, thereby making multiple-dynein-driven motion along a single MT quite unlikely. Similar conclusions have been reached using computer simulations (Erickson et al., 2011). This problem can be overcome by generating a nonuniform dynein distribution, with multiple dyneins clustered within *A*_*CONTACT*_. We emphasize that activating dynein molecularly using regulatory proteins (Vallee et al., 2012) is of little help, because the activated dyneins would never be able to contact a single MT simultaneously without clustering.

### Dynein, Flotillin, and Rab7 Colocalize into Microdomains on the Phagosome Membrane

**Figure S3:**
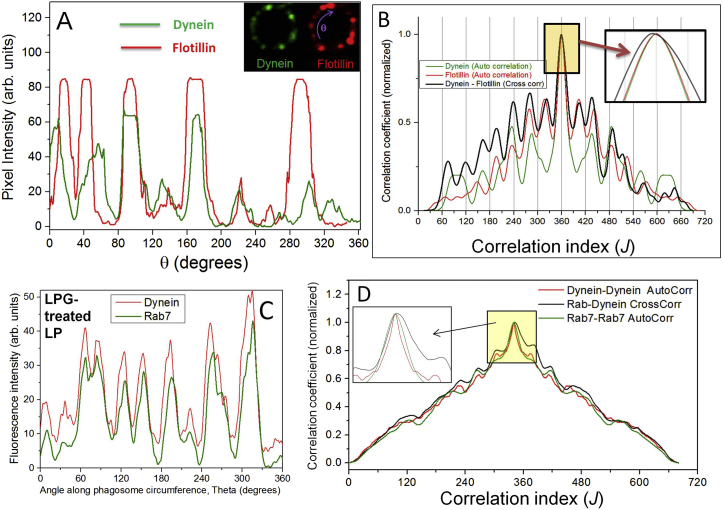
Colocalization of Dynein and Rab7 on Late Phagosomes: Effect of LPG on Fluorescence Intensity Patterns of Dynein and Rab7 on Late Phagosomes, Related to Figures 3 and 5 (A) Inset: Confocal fluorescence image of a single late phagosome (LP) that was double immunostained for dynein and flotillin. Fluorescence intensity was measured for dynein (green channel) and flotillin (red channel) as a function of angular rotation (θ; indicated). Fluorescence intensity along circumference is plotted as a function of the angular position along LP circumference for dynein and flotillin. Note how the peaks (position of puncta) occur at the same value of Ө, suggesting colocalization. (B) Cross correlation analysis was done to investigate co-localization of dynein and flotillin along the full circumference (360 degrees) of an LP. Fluorescence intensity profiles shown in Figure S3-A were used. Further details can be found in Section 15 of Supplemental Experimental Procedures. The maximum value of the cross-correlation coefficient (black) almost coincides with the maximum of both autocorrelation coefficients (green and red; see inset). This supports the close matching (within a few degrees) between dynein and flotillin-1 staining, and therefore the colocalization of these two proteins on phagosome membrane. As expected, the autocorrelation patterns (green and red lines) are exactly symmetric about their peak at 360 degrees. The cross-correlation pattern does not have this exact symmetry, and is wider than the autocorrelation peaks (see inset) because the dynein and flotillin stainings do not match exactly. (C) The fluorescence intensity profiles for dynein and Rab7 on an LPG-treated LP. The intensity profiles were obtained by tracing a circle around the LP for dynein as well as Rab7 channels. Note the close matching between red and green patterns, suggesting that dynein and Rab7 continue to colocalize after LPG treatment. (D) Computation of the cross correlation coefficient between intensity profiles of dynein and Rab7 on LP1 (shown in Figure S3-C). The maximum value of the cross-correlation coefficient (black) coincides with the maximum of both autocorrelation coefficients (green and red; see inset). This suggests that dynein intensity pattern closely overlaps with Rab7 pattern. We therefore believe that dynein and Rab7 continue to colocalize after LPG treatment. A more rigorous analysis of the colocalization was not attempted because of the diffraction limited nature of these images.

**Figure S4:**
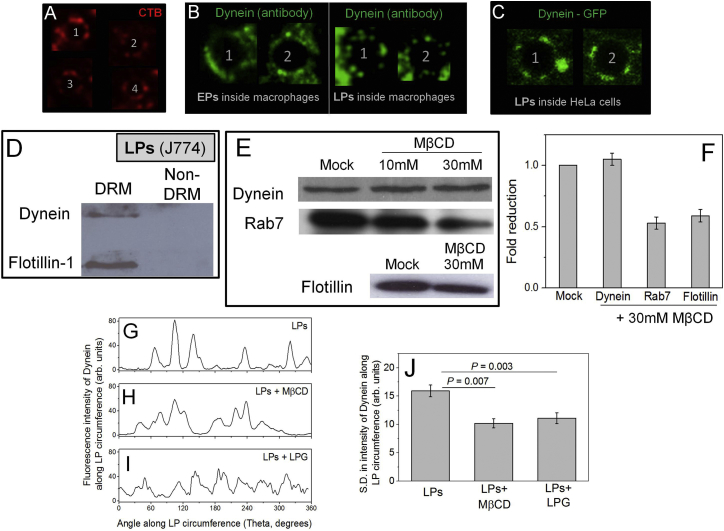
Cholera Toxin and Dynein Staining on Late Phagosomes, Appearance of Dynein in DRM Fraction, and Effect of MβCD and LPG on Proteins Associated with Late Phagosomes, Related to Figures 3, 4, and 5 (A) LPs (numbered 1-4) inside J774 cells were stained against Alexa Fluor 594 conjugated Cholera Toxin Subunit B (CTB) and visualized in a confocal microscope. Staining around the LP circumference is seen, supporting the presence of cholesterol-rich microdomains/lipid rafts on LPs. LP diameter = 2 microns. (B) Immunofluorescence staining of EPs (left panel) and LPs (right panel) against dynein inside mouse macrophage cells. The staining on EP circumference is more uniform compared to the punctate staining on LPs. Two EPs from different cells (marked 1,2) and two LPs from different cells (marked 1,2) are shown. EP and LP diameter = 2 microns. (C) Staining of two LPs (marked 1,2) inside HeLa cells expressing Dynein-GFP (see text). No antibody is used. Dynein again appears as puncta on the LP surface. LP diameter = 2 microns. (D) LPs purified from J774 cells were used to isolate detergent resistant membrane (DRM) and non-DRM fractions. Western blotting was performed to probe for presence of dynein intermediate chain and flotilllin. Both proteins are detected in the DRM fraction, but not detected in the non-DRM (soluble) fraction. This experiment was repeated thrice with similar results. (E) Western blots against dynein, Rab7 and flotillin-1 as a function of MβCD concentration on LPs purified from J774 cells. (F) Quantification of western blot intensities in Figure S4-E shows that there is no reduction in the amount of dynein upon MβCD treatment, but ∼50% reduction in Rab7 and flotillin-1. Error bars are SEMs. Dynein has a secondary binding site to late endosomes/lysosomes via the dynein light intermediate chain (LIC1) that is independent of Rab7-RILP-dynactin. It is therefore possible that dynein is retained through this mechanism even after cholesterol and Rab7 are lost after MβCD treatment. (G–I) Fluorescence intensity profile along the circumference of an untreated LP, an MβCD-treated LP and an LPG-treated LP. Sharp peaks for dynein staining on untreated LPs are replaced by broader features after MβCD and LPG treatment. (J) Standard deviation (SD) of fluorescence intensity profile measured for multiple LPs along their circumference (10 each of untreated, MβCD treated and LPG treated LPs used). SD was reduced significantly after MβCD and LPG treatment, suggesting that the clustered organization of dynein was replaced by a more uniform distribution. This was also verified by a cross correlation analysis of these intensity profiles. A more rigorous analysis of the disruption in clustering was not attempted because of the diffraction limited nature of these images.

What mechanisms can cluster dynein on a cargo membrane? The staining of dynein on LPs (Figure 3A) was reminiscent of similarly punctate staining for the cholesterol-binding protein flotillin on phagosomes (Dermine et al., 2001). We therefore suspected that dynein localizes into, and clusters within, cholesterol-rich microdomains on the LPs. Double-immunostaining of LPs purified from J774 cells against dynein and flotillin showed that dynein indeed co-localizes with flotillin into punctate structures on LPs (Figure 3F; two representative LPs numbered 1 and 2 are shown). This is also clear from the overlapping pixel intensity profiles of dynein and flotillin along the circumference of an LP (Figure S3A). Cross-correlation analysis also confirmed the co-localization of dynein with flotillin (Figure S3B). LPs also showed a punctate staining for cholera toxin B as reported earlier (Dermine et al., 2005), confirming the presence of lipid microdomains on LPs (Figure S4A).

The GTPase Rab7 interacts with dynein and recruits dynein to LPs in a GTP-dependent manner (Harrison et al., 2003, Rocha et al., 2009). Rab7 also interacts with the cholesterol sensor ORP1L (Rocha et al., 2009) and could therefore be present in membrane microdomains. Double immunostaining for dynein and Rab7 showed that these proteins colocalize into the same punctae on LPs (Figure 3G). In contrast to dynein and Rab7, distribution of the LP marker LAMP1 was uniform and continuous (Figure 3H), as also reported by others (Dermine et al., 2001). Figure 3I plots the SD (i.e., the fluctuation) in fluorescence intensity calculated along the circumference of LPs for dynein, flotillin, and LAMP-1 (five LPs used for each protein). The punctate staining of dynein and flotilin-1 is reflected in significantly higher SD compared to LAMP1.

To investigate whether dynein’s punctate staining is an artifact from disruption of phagosome membrane during purification, we performed immunostaining for dynein inside macrophage cells. While the staining on EPs was more uniform, a punctate staining for dynein was again apparent on LPs inside cells (Figure S4B). To investigate whether punctate staining is an artifact of antibody clustering, we phagocytosed latex beads into stable HeLa bacterial artificial chromosome (BAC) cells where the dynein intermediate chain is tagged to GFP (Poser et al., 2008). Dynein-GFP again appeared in punctate arrangement on LPs (Figure S4C). We also isolated detergent-resistant membranes (DRMs) from purified LPs (Goyette et al., 2012) to find that dynein is enriched in the DRM fraction along with flotillin-1 (Figure S4D). However, DRM formation may be an artifact and its relevance to lipid microdomains inside cells is unclear (Rao and Mayor, 2014).

### Increase in Membrane Cholesterol on Phagosomes with Maturation

**Figure 4:**
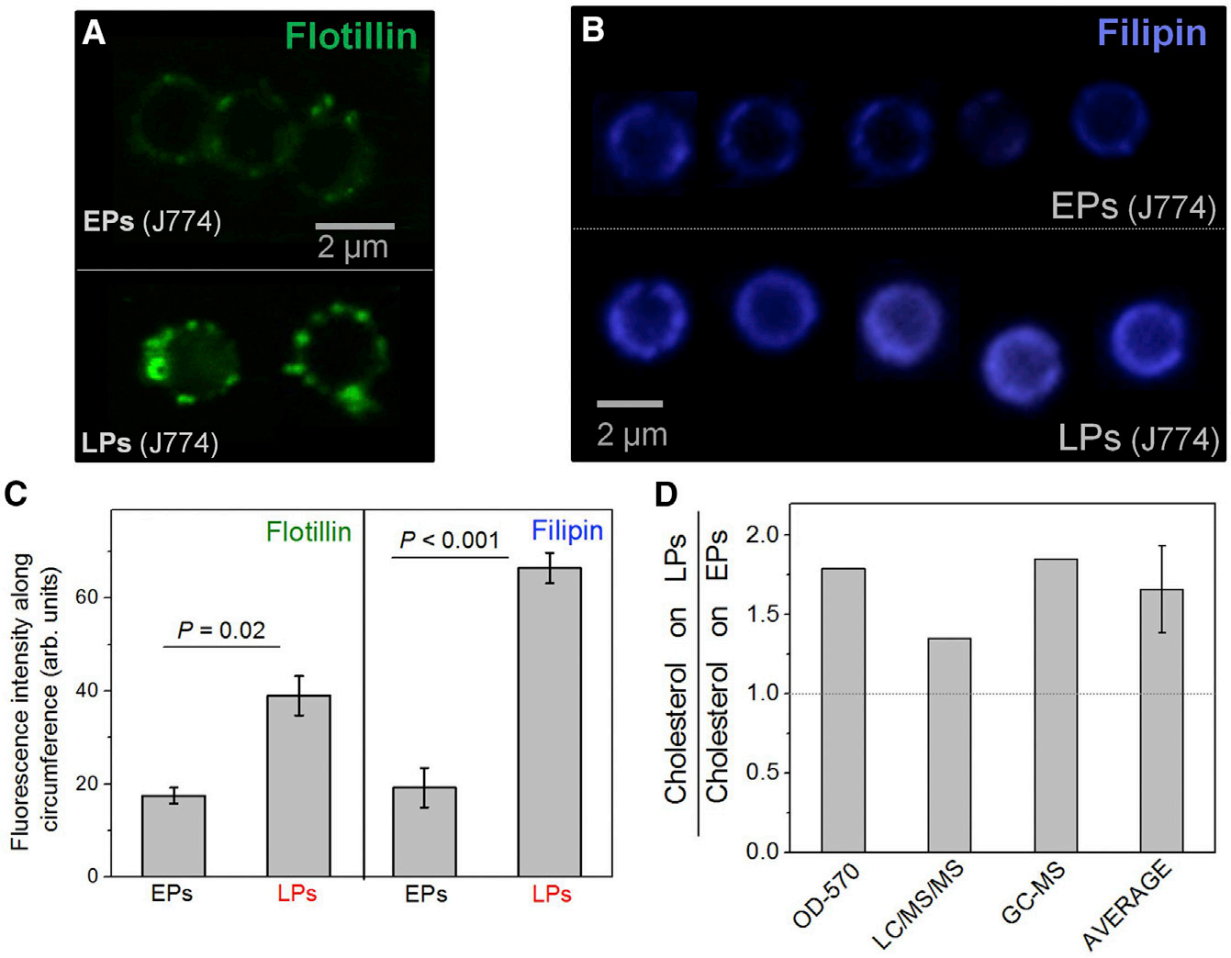
LPs Have More Membrane Cholesterol Than EPs (A) Confocal immunostaining images of purified EPs and LPs using antibody against flotillin-1. (B) Representative image of purified EPs and LPs stained for filipin (a cholesterol marker). Images are taken under epifluorescence illumination. (C) Mean intensity of flotillin and filipin staining along circumference for EPs (10 used) and LPs (49 used). Error bars represent SEM. (D) Ratio of cholesterol on LPs to EPs, as measured by different methods (see main text). OD-570, measurement of cholesterol by colorimetry using an Abcam assay kit. LC/MS/MS, liquid chromatography mass spectrometry. GC-MS, gas chromatography mass spectrometry for cholesterol. The average of all measurements is also shown (error bar represents SD). See also Figures S4, S5, and S6.

Since cholesterol is an important component of membrane microdomains, we investigated whether clustering of dynein correlates with an increase in membrane cholesterol on LPs. This possibility is supported by the enrichment of the cholesterol-binding protein flotillin on late phagosomes (Goyette et al., 2012). Immunofluorescence images revealed barely detectable flotillin-1 on EPs, but intense and punctate staining on LPs (Figure 4A). We next stained purified EPs and LPs with filipin, an antibiotic used to detect cholesterol in lipid membranes. Images for filipin could only be acquired under epifluorescence illumination, possibly obscuring the punctate staining of filipin on LPs. Higher filipin staining was observed on LPs as compared to EPs (Figure 4B). A statistically significant increase in pixel intensity of flotillin and filipin was measured on the LP circumference (Figure 4C). The images in Figures 4A and 4B provide microscopic evidence for higher cholesterol on LPs as compared to EPs.

**Figure S5:**
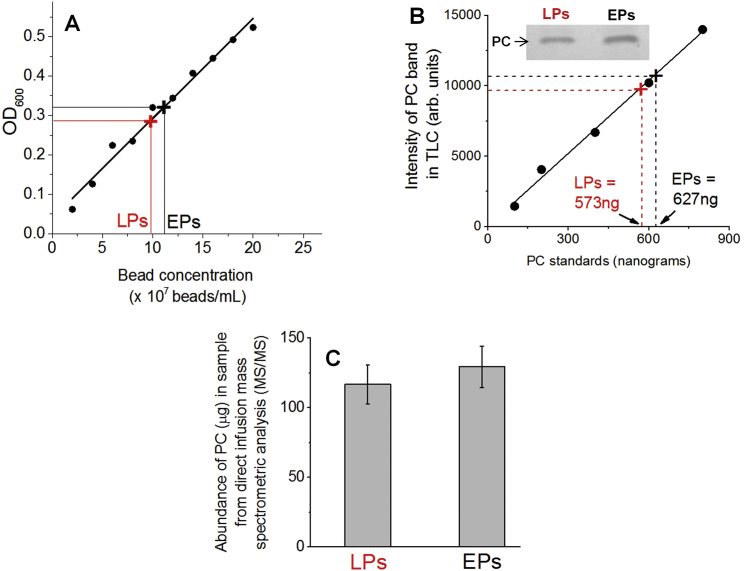
Experiments to Obtain EP and LP Samples with Equal Number of Phagosomes, Related to Figures 4 and 5 (A) The optical density at 600nm (OD_600_) as a function of bead concentration for latex bead phagosomes. OD_600_ varies linearly with bead concentration, and can therefore be used to normalize two phagosome preparations (e.g., an EP sample and an LP sample). A similar curve could be obtained using silica beads using OD_400_. The observed values of OD_600_ for an EP and an LP sample are indicated. There appears to be slightly larger number of phagosomes in the EP sample based on OD. (B) These EP and LP samples were subjected to thin layer chromatography (TLC). The band for phosphatidylcholine (PC) is shown (upper inset). A TLC experiment was also run for PC samples with known amounts of pure PC (standards). The intensity of PC band for known standards is plotted. This is used as a calibration curve to obtain the actual amount of PC in the EP and LP samples (indicated). As expected, the EPs have slightly more PC. (C) These EP and LP samples were then subjected to direct infusion - mass spectrometry (MS/MS) for analysis of PC. The abundance of PC is plotted, and again shows that the EP sample has slightly more PC than LP. This is consistent with the OD and TLC data (panels A and B). These samples were then adjusted by dilution to obtain an EP and an LP sample with equal amounts of PC (and therefore with equal number of phagosomes having equal membrane content because an EP and an LP have the same uniform size).

**Figure S6:**
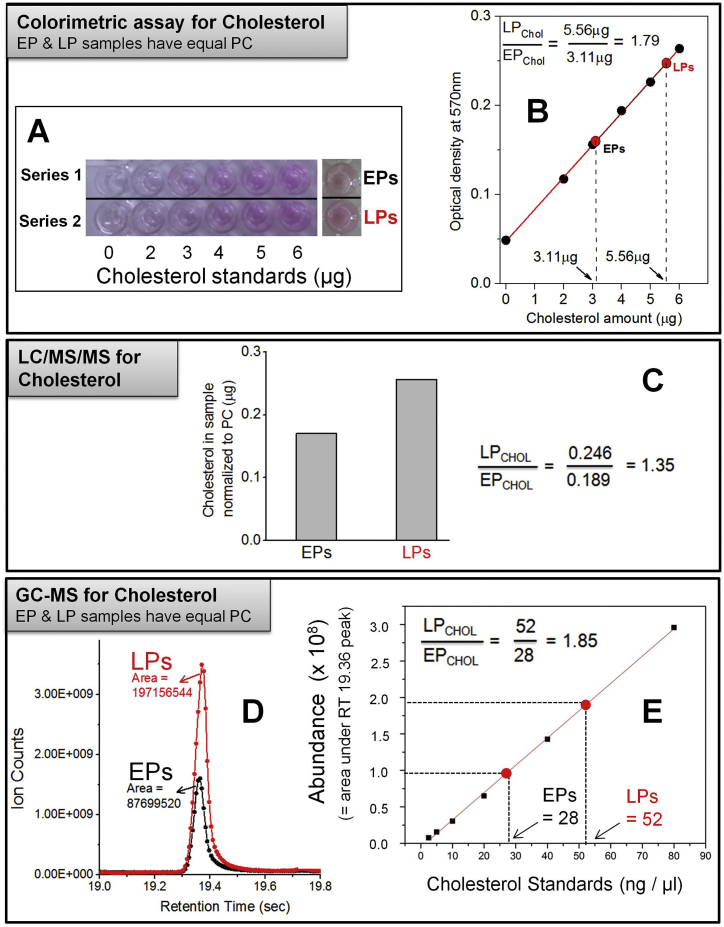
Cholesterol Estimation on Phagosomes, Related to Figures 4 and 5 (A) Known cholesterol standards were prepared by dilution. Two sets of identical dilutions (Series1, Series2) were used to determine OD at 570nm. The average OD_570_ at a particular cholesterol dilution was determined from these two series. The EP and LP samples (having equal PC) are also shown on the right. The difference in color (i.e., difference in cholesterol) between EPs and LPs is obvious from this image. The EP and LP images have a darker background compared to the cholesterol standards because the camera flash did not illuminate all samples uniformly when this picture was taken. This has no bearing on the measurements of individual wells done in a plate reader. See section 12 of Supplementary Methods for more details. (B) The average of OD_570_ (Series1+Series2)/2 is plotted as a function of the (known) cholesterol content of these samples. A linear increase in OD_570_ can be seen with increasing cholesterol. The red line is a linear fit. The OD_570_ measured for EPs and LPs (red circles) corresponds to amounts of cholesterol mentioned on the *X*-axis. The ratio of cholesterol on LPs:EPs is also calculated ( = 1.79; top inset). (C) EP and LP samples were subjected to LC/MS/MS to measure free cholesterol and PC. Measurements were done at Avanti Polar Lipids. The amount of cholesterol (after normalizing for PC) is plotted. The ratio of cholesterol (LPs:EPs) is also calculated. (D) EP and LP samples (having equal PC) were subjected to GC-MS to measure the amount of free cholesterol. The peak for cholesterol is shown. Calculated area under the peak is mentioned. (E) A calibration curve was prepared by GC-MS using known cholesterol standards. The amount of cholesterol in EP and LP samples was calculated using this calibration curve (calculated amounts mentioned in figure). The LP:EP cholesterol ratio is also calculated (top). Note that the calibration curve is linear and values of cholesterol on EPs and LPs fall within the linear range.

We next estimated the amount of membrane-associated (free) cholesterol on bulk samples of purified EPs and LPs. This was done using a cholesterol assay kit and also by quantitative lipidomics (Figures S5 and S6; see Supplemental Experimental Procedures, section 12). Averaging over all these methods, we found ∼1.6-fold more cholesterol on LPs than on EPs (Figure 4D). Sphingomyelin (SM) and cholesterol are known to interact, and their concentrations correlate in cell membranes (Ito et al., 2000). The SM content is 1.74 times higher in LPs than EPs (Desjardins et al., 1994). This is in good agreement with the LP:EP cholesterol ratio measured here. Since EPs are derived from the cholesterol-rich plasma membrane, it is not obvious how LPs acquire more cholesterol than EPs. It is possible that EPs are formed from cholesterol-poor domains of the plasma membrane. A role for cholesterol recycling from endosomes is also possible, though these issues remain to be addressed in detail.

### Dynein Clustering Hypothesis for Rapid Unidirectional Transport of Late Phagosomes

The observed increase in cholesterol and punctate staining of dynein/Rab7/flotillin on LPs (Figures 3A, 3F, and 3G) prompted us to make the following dynein clustering hypothesis: As phagosomes mature, they fuse with cholesterol-rich endolysosomes to acquire cholesterol and membrane associated “raftophilic” proteins (e.g., Rab7, flotillin) that promote and stabilize microdomain formation on the LP membrane. Rab7 interacts with cholesterol-bound ORP1L and is therefore recruited preferentially within cholesterol-rich microdomains on LPs. Rab7-ORP1L binding stabilizes a GTP-bound state of Rab7, which in turn recruits Rab-interacting lysosomal protein (RILP), dynactin, and dynein to the microdomains on LPs (Rocha et al., 2009). ORP1L-Rab7-RILP bound dyneins therefore cluster within microdomains. Multiple dyneins clustered within a microdomain can simultaneously engage a single MT to generate robust directed transport of LPs.

Blocking the vacuolar proton pump ATPase with bafilomycin prevents fusion of phagosomes with late endosomes and also blocks the recruitment of flotillin-1 to late phagosomes (Dermine et al., 2001). Cholesterol is therefore likely acquired by phagosomes via interactions with the endosome membrane. Rab7 may also have a role in regulating dynein function on LPs, but we do not believe that it directly regulates dynein’s single molecule function. Rather, Rab7 possibly first localizes to microdomains using its lipid anchor and then acts as a local scaffold to recruit dyneins preferentially within the microdomains. It is also likely that Rab7-GTP stabilizes the formation of microdomains, because Rab7-GTP can dimerize on the lipid membrane (Johansson et al., 2007). We are unable to comment on the possible co-clustering of kinesin with dynein into microdomains or the exclusion of kinesin from microdomains. Kinesin-1 could not be detected on LPs by immunofluorescence and western blots of DRM fractions, possibly because there is ∼20-fold less kinesin-1 compared to dynein on LPs (Rai et al., 2013). It also appears more physiologically relevant to cluster dynein into microdomains, because dynein (and not kinesin) is adapted to work in large teams (Mallik et al., 2013, Rai et al., 2013).

### Effect of Cholesterol Depletion on Late Phagosomes

**Figure 5:**
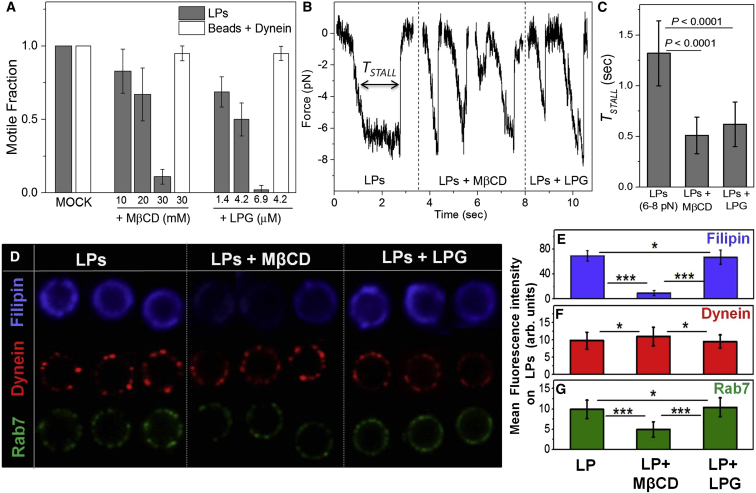
Effect of Cyclodextrin and Lipophosphoglycans on Dynein’s Function and Organization on LPs (A) Motile fraction of dynein-coated beads and LPs treated with MβCD and lipophosphoglycans (LPGs) purified from *Leishmania*. There is a dose-dependent reduction of LP motion upon MβCD and LPG treatment. Such treatments had no effect on motility of dynein-coated beads. Error bars represent SD. (B) Minus-end-directed stall force records of LPs that were mock treated, MβCD treated (10 mM), or LPG treated (4.2 μM). The maximum force is ∼7 pN in all cases (i.e., six or seven dyneins). Plateau-like region for untreated LPs is absent for MβCD and LPG treated LPs. (C) *T*_*STALL*_ calculated from stalls such as shown in Figure 5B. *T*_*STALL*_ is significantly reduced after MβCD and LPG treatment of LPs. Error bars represent SD. (D) Representative images of untreated, MβCD treated (10 mM), or LPG treated (4.2 μM) LPs stained for filipin (cholesterol marker), dynein, and Rab7. Images for filipin acquired under epifluorescence illumination. Images for dynein and Rab7 were acquired on a confocal microscope. (E) Reduction in fluorescence intensity of filipin on LPs after MβCD treatment, but no reduction after LPG treatment. A minimum of 20 LPs was used for each case. Analysis performed in blind manner for (E)–(G). Error bars represent SEMs. ^∗^p > 0.2, ^∗∗∗^p < 0.001. (F) No reduction in fluorescence intensity of dynein is seen along the circumference of LPs after MβCD or LPG treatment. A minimum 20 LPs was used for each case. Error bars represent SEM. (G) A statistically significant reduction in fluorescence intensity of Rab7 along the circumference of LPs is seen after MβCD treatment. No such reduction is seen after LPG treatment. A minimum 20 LPs was used for each case. Error bars represent SEM. See also Figures S4, S5, and S6.

To verify a role for cholesterol in dynein-driven LP motion, we performed in vitro motility assays with purified LPs after incubation with methyl-beta-cyclodextrin (MβCD), which removes cholesterol from lipid membranes. MβCD did not influence dynein’s molecular function, because beads passively coated with purified dynein showed no change in velocity and motile fraction after addition of MβCD (Figure 5A). In contrast, motile fraction of dynein driven LPs was significantly reduced after MβCD treatment (Figure 5A). MβCD had no effect on velocity of the few minus-end-directed LPs that moved (2.1 ± 0.3 μm/s at 20 mM MβCD, similar to untreated LPs). These observations suggest that MβCD disrupts transport of LPs without interfering with the single-molecule function of dynein. We observed infrequent minus-end-directed stalls of LPs with lower forces after MβCD treatment (Figure 5B). The plateau-like regions usually seen for LPs were missing, and *T*_*STALL*_ was significantly lowered (Figure 5C). Multiple dyneins therefore failed to generate force cooperatively and detached abruptly against load after MβCD treatment. A reduction in filipin staining confirmed that MβCD removed cholesterol from LP membranes (Figures 5D and 5E). The sharp punctate staining of dynein on LPs subtly changed to a more diffuse distribution after MβCD treatment (Figure 5D; see quantification later). Interestingly, the mean intensity for dynein staining along circumference was unchanged (Figure 5F), suggesting no loss of dynein from LPs upon MβCD treatment. This was verified by western blotting experiments, where no change in dynein amount on purified LPs was seen after MβCD treatment (Figures S4E and S4F). Analysis of the fluctuations in fluorescence intensity also suggested a more diffuse distribution of dynein after MβCD treatment of LPs (Figures S4G, S4H, and S4J).

Since MβCD did not remove dynein from LPs and did not interfere with motion of dynein-coated beads, it could inhibit LP motion by disrupting dynein clusters or by removing dynein-associated regulatory proteins from the LP membrane. Indeed, we detected reduced intensity of Rab7 and flotillin staining on LPs after MβCD treatment (Figure 5D). Approximately 50% reduction in Rab7 and flotillin was estimated by measuring fluorescence intensity on treated and untreated LPs (29 each) and by performing western blots of purified LPs (Figure S4F). MβCD experiments therefore suggested a role of cholesterol in dynein-driven LP motility but could not distinguish between two possible mechanisms of motility reduction, namely disruption of dynein’s clustered organization versus loss of dynein regulators (e.g., Rab7). To address this issue, we next devised experiments to disrupt the microdomains on LPs without removing cholesterol and Rab7 from LPs.

### Effect of Leishmania Lipophosphoglycans on Late Phagosomes

The protozoan parasite *Leishmania donovani* causes visceral Leishmaniasis or *kala-azar*, the second largest parasitic killer disease in the world after malaria. *Leishmania* targets a liver-specific microRNA to reduce serum cholesterol, and liposomal delivery of cholesterol can protect against leishmaniasis (Ghosh et al., 2013). This suggests that cholesterol is a major player in *Leishmania* pathogenesis. The *Leishmania* promastigote resides within phagosomes inside macrophages but avoids degradation by altering its fusion with late endosomes and lysosomes (Dermine et al., 2000, Dermine et al., 2005). Similar to LPs, flotillin has been observed in punctate arrangement on *Leishmania*-containing phagosomes (Dermine et al., 2001). *Leishmania* expresses a cell-surface glycolipid called lipophosphoglycan (LPG). Studies with mutant *Leishmania* lacking LPG show that LPG is the main molecule that blocks fusion of *Leishmania* with lysosomes. LPG is a GPI-anchored glycolipid with a glycan core and a polymer of Gal(β 1,4)Manα1-PO_4_ repeating units. The GPI lipid anchor presumably localizes LPG to cholesterol-rich domains on the surface of *Leishmania*-containing phagosomes. Western blot and slot blot experiments showed no change in amount of flotillin and GM1 on phagosomes after LPG treatment—this suggests that LPG treatment may not remove microdomain-associated proteins (e.g., Rab7, flotillin) from the phagosome membrane (Dermine et al., 2005).

We therefore assayed minus-end-directed motility of LPs in presence of LPG to observe a reduction in the motile fraction after LPG treatment (Figure 5A). The few LPs that moved in presence of LPG showed velocity equivalent to untreated LPs (2.0 ± 0.4 μm/s), suggesting that enzymatic function of dynein is not perturbed by LPG. We next measured cooperative force generation by dynein on LPs in presence of LPG. Infrequent stalls at lower force (<8 pN) were observed, with the plateau-like region typical of untreated LPs absent (Figure 5B; compare stalls of ∼7 pN for LPG-treated and untreated LPs). This was also evident from the significantly lower values of *T*_*STALL*_ after LPG treatment (Figure 5C). Motility and cooperative force generation of LPs was therefore inhibited by LPG in a manner similar to MβCD (Figures 5A–5C). Similar to the experiments with MβCD, LPG had no effect on motile fraction (Figure 5A) and velocity of beads coated with dynein. This suggests that LPG does not inhibit dynein’s single-molecule function.

Figure 5D (top, right) shows filipin staining of LPG-treated LPs, and Figure 5E plots the mean filipin intensity measured along circumference of untreated and LPG-treated LPs (49 and 31 LPs used, respectively). No reduction in filipin staining was seen, suggesting that LPG does not remove cholesterol from the phagosome membrane. This observation agrees with reports that LPG does not remove flotillin (a cholesterol-binding protein) from phagosomes (Dermine et al., 2005). If cholesterol is not removed, cholesterol-associated proteins may also not be removed after LPG treatment. This was indeed suggested by the unchanged total intensity of dynein and Rab7 staining on LPs after LPG treatment (Figure 5D). Quantification of dynein and Rab7 fluorescence intensity along circumference of LPs also confirmed that dynein (Figure 5F) and Rab7 (Figure 5G) are not removed after LPG treatment. While LPG did not remove dynein and Rab7, the punctate staining of these proteins changed to a more uniform distribution after LPG treatment (compare untreated versus LPG-treated LPs in Figure 5D). Analysis of the fluctuations in fluorescence intensity also suggested a more diffuse distribution of dynein after LPG treatment of LPs (Figure S4G, S4I, and S4J). A cross-correlation analysis suggested that dynein and Rab7 still continue to colocalize on the LP membrane after LPG treatment (Figures S3C and S3D). These experiments suggest that LPG does not remove but merely redistributes microdomain-associated proteins (like dynein and Rab7) from clustered to more uniform organization on phagosomes. We therefore conclude that robust long-distance transport of LPs by dyneins is primarily caused by the cholesterol-dependent geometrical clustering of dynein into microdomains.

## Discussion

**Figure 6:**
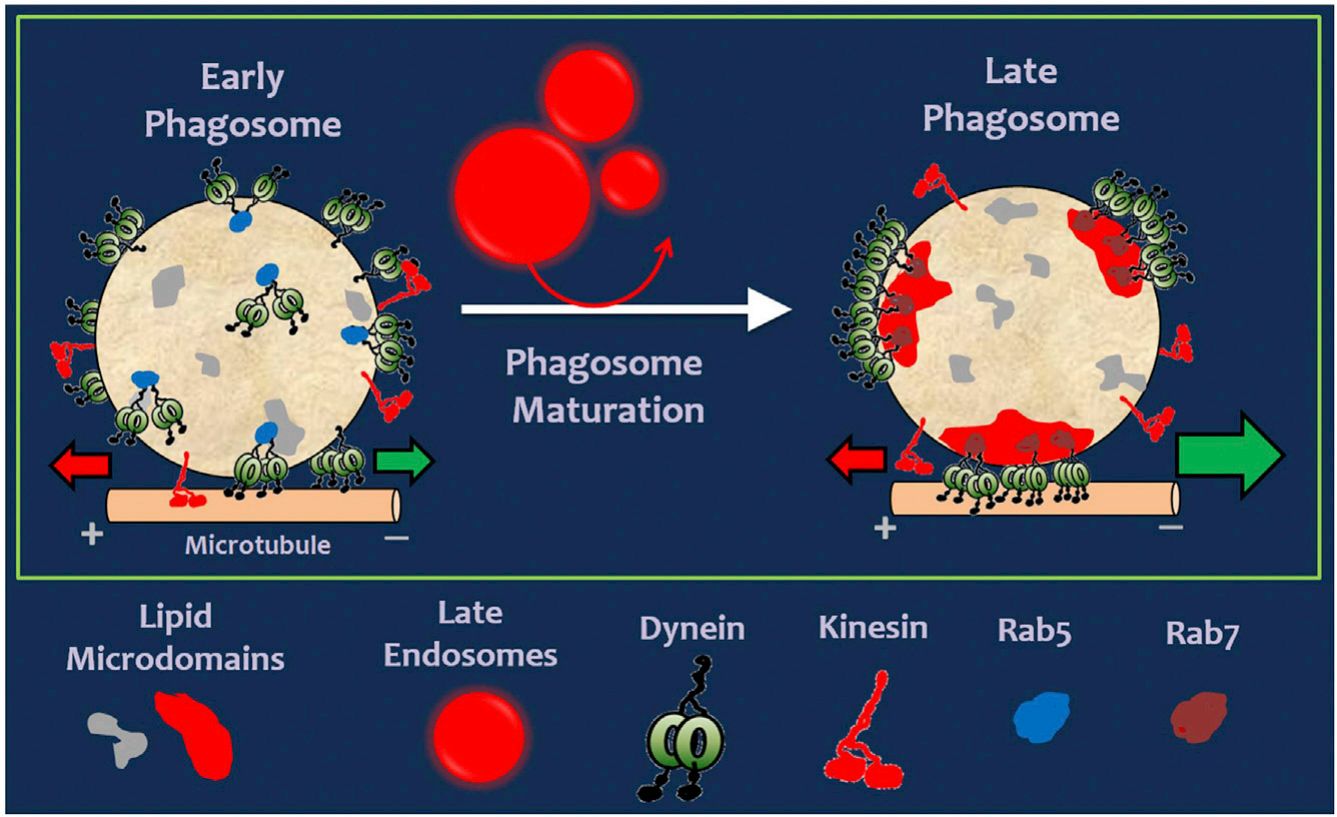
Model for the Bidirectional to Retrograde Switch in Phagosome Transport An early phagosome with dynein and kinesin motors is shown. Motors are randomly distributed on the surface. Some dyneins are shown engaged to a MT at the bottom through Rab5. A kinesin is also engaged to the MT. This situation results in force balanced bidirectional motion interspersed by a tug of war between kinesin and dynein (opposing red and green arrows of equal size). Phagosomes acquire membrane cholesterol as they mature because of physical interactions with cholesterol-rich late endosomes (red spheres). Late phagosomes now develop stable cholesterol-rich microdomains (three microdomains are shown). Microdomain formation is likely facilitated by Rab7-GTP. Dynein, along with Rab7, clusters into microdomains to form “force-generating platforms” where multiple dyneins are in close proximity and ordered orientation. This generates large persistent force to drive minus-end-directed transport of the phagosome (large green arrow). Kinesin may possibly be excluded from such microdomains (remains to be investigated). Note that the total number of motors (dynein or kinesin) remains almost unchanged between EPs and LPs.

Dynein-driven transport promotes physical interactions between phagosomes and endolysosomes. This likely enables pathogen clearance by allowing phagosomes to acquire microbicidal properties and low pH. We show here that an increase in cholesterol and cholesterol-associated proteins (e.g., Rab7 and flotillin) clusters dynein within cholesterol-rich microdomains to assemble dynein teams on the phagosome membrane. Once clustered, many dyneins within a microdomain can simultaneously engage a single MT to bias the transport of LPs in the minus direction (toward lysosomes). This mechanism of geometrical clustering is very different from the widely discussed single-molecule regulation of motors by regulatory proteins (Vallee et al., 2012, Verhey and Hammond, 2009). The proposed clustering mechanism is schematized in Figure 6 and appears primarily responsible for the bidirectional-to-retrograde switch in motion during phagosome maturation. Because micron-sized cellular cargoes are common, clustering of motors may have general relevance in regulating intracellular transport. However, the mechanism of clustering could be cargo specific. Cholesterol also enhances transport of artificial liposomes by kinesin-3 (Klopfenstein et al., 2002), which is a monomeric motor in mammals (Soppina et al., 2014) and binds to the phospholipid PtdIns(4,5)P_2_. Cholesterol induces clustering of PtdIns(4,5)P_2_, and therefore of kinesin-3, whereupon the monomers assemble into a dimeric kinesin-3 that is highly processive. Thus, a cooperative increase in kinesin-3-driven transport is observed. Unlike monomeric mammalian kinesin-3, native cytoplasmic dynein exists as a homodimer of heavy chains in all known organisms (Vallee et al., 2012). A monomer-dimer transition (like kinesin-3) is therefore ruled out for dynein, and the improved minus-end-directed LP motion must arise from clustering of inherently dimeric dyneins into microdomains.

What are the advantages of clustering dynein on a cellular cargo? Clustering should favor directed linear motion along a single MT by preventing simultaneous engagement of dynein present all over the cargo with multiple randomly oriented MTs. Membrane microdomains are also suggested to support, confine, and redirect force within the lipid membrane by behaving as a mechanically stiff platform (Anishkin and Kung, 2013). It is therefore possible that phagosomal microdomains behave as force-generating platforms, on which force from multiple dyneins can be oriented and directed more effectively. Earlier reports show a gear-like behavior in dynein (Mallik et al., 2004) and a large collective force by dynein teams on LPs inside cells (Rai et al., 2013), perhaps facilitated by this gear-like behavior. Here, we show that geometrical clustering into microdomains assembles dynein teams to facilitate such large forces (Figure 6). A hierarchy of cellular mechanisms therefore appears to harness dynein function for a crucial biological process, namely endophagosome maturation and degradation of pathogens. There exists a vast literature on the mechanisms of membrane microdomain (lipid raft) formation in cells and implications thereof. However, the downstream biological consequences of such microdomain formation have remained elusive. Our work brings out an experimentally observable, direct functional consequence of lipid microdomain formation to intracellular transport and phagosome/pathogen biology.

## Experimental Procedures

Phagosomes created by phagocytosing 759-nm-diameter latex beads were observed using differential interference contrast microscopy (Barak et al., 2013, Barak et al., 2014). For further details, see sections 3 and 4 of Supplemental Experimental Procedures. Phagosome motion was visualized inside agar-flattened *Dictyostelium* cells (section 5, Supplemental Experimental Procedures). Purification and in vitro motility of latex bead phagosomes has been described (Barak et al., 2014). Further details can be found in Supplemental Experimental Procedures (section 6). Phagosomes were prepared using silica beads or latex beads from J774, RAW264.7, or *Dictyostelium* cells. Purity of latex bead phagosomes was confirmed using markers against endosomal, cytosolic, and membrane proteins (Supplemental Experimental Procedures, section 6; Figure S2). Confocal imaging was used to detect proteins on the phagosome membrane. EPs/LPs were treated with filipin and imaged under epifluorescence illumination. Further details can be found in Supplemental Experimental Procedures, section 7 (for phagosomes from J774 and RAW cells) and section 9 (for phagosomes from *Dictyostelium*). Measurement of fluorescence intensity on phagosomes is described in Supplemental Experimental Procedures, section 8. Statistical hypothesis testing was done using Student’s t test. Two-tailed p values (95% confidence) were calculated. Error bars are SD or SEM, as indicated.

DRM isolation from purified phagosomes was done as described previously (Goyette et al., 2012). Further details can be found in in section 11 of Supplemental Experimental Procedures. Lipids were extracted from phagosomes using a methanol-chloroform mixture for thin-layer chromatography (TLC) experiments. Silica TLC plates were used to separate the lipids with an appropriate solvent system, followed by visualization on a Bio-Rad instrument. Further details can be found in section 12 of Supplemental Experimental Procedures. MβCD prepared in buffer (30 mM Tris and 4 mM EGTA [pH 8.0]) was incubated with LPs (22°C, 15 min) at final concentrations ranging from 10 mM to 30 mM. Further details can be found in section 13 of Supplemental Experimental Procedures. LPG purified from *Leishmania donovani* (Turco et al., 1987) was obtained as a gift. The stock solution (in ddH_2_O) was diluted appropriately. LPs were incubated with LPG (22°C, 15 min) before observation (Dermine et al., 2005). Further details can be found in section 13 of Supplemental Experimental Procedures. Bead motility with dynein using an ATP releasate from *Dictyostelium* cells has been described elsewhere (Soppina et al., 2009b). Further details can be found in section 14 of Supplemental Experimental Procedures. See Supplemental Experimental Procedures, section 12 for details of lipidomics measurements. PC and free cholesterol was measured on lipids obtained from EPs and LPs purified from RAW264.7 cells.

## Author Contributions

A.R., D.P., S.T., S.S., A.K.D., and R.M. performed research and analyzed data. A.R., D.P., and R.M. wrote the paper. R.M. designed the research.

## Supplemental Information

Document S1. Supplemental Experimental Procedures

Movie S1. Motion of an Early Phagosome Inside an Agar-Flattened Dictyostelium Cell, Related to Figure 1 Movie runs in real time. Initial position of a motile early phagosome (EP) is circled. Various other organelles can also be seen moving vigorously inside the cell.

Movie S2. In Vitro Motion of an Early Phagosome Purified from *Dictyostelium* along a Polarity-Labeled Microtubule, Related to Figure 1 Movie runs in real time. The microtubule is not visible because it out of focus and has low contrast. magnetic beads labeling minus end of microtubule are indicated. The early phagosome (EP) moves bidirectionally (in back-and-forth manner; indicated by double headed red arrow) on the MT. An EP that is stuck on the MT is also shown. The EP is 759nm in diameter.

Movie S3. In Vitro Motion of a Late Phagosome Purified from *Dictyostelium* along a Polarity-Labeled Microtubule, Related to Figure 1 Movie runs in real time. The microtubule is not visible because it out of focus and has low contrast. Magnetic beads labeling minus end of microtubule are indicated. The late phagosome (LP) moves unidirectionally towards the minus end of the MT (indicated by red arrow). Another LP that had been placed on the same MT has moved to reached the extreme minus end of MT and is now stuck there. The LP is 759nm in diameter.

Document S2. Article plus Supplemental Information

## References

[bib1] Anishkin A., Kung C. (2013). Stiffened lipid platforms at molecular force foci. Proc. Natl. Acad. Sci. USA.

[bib2] Barak P., Rai A., Rai P., Mallik R. (2013). Quantitative optical trapping on single organelles in cell extract. Nat. Methods.

[bib3] Barak P., Rai A., Dubey A.K., Rai P., Mallik R. (2014). Reconstitution of microtubule-dependent organelle transport. Methods Enzymol..

[bib4] Blocker A., Severin F.F., Habermann A., Hyman A.A., Griffiths G., Burkhardt J.K. (1996). Microtubule-associated protein-dependent binding of phagosomes to microtubules. J. Biol. Chem..

[bib5] Blocker A., Severin F.F., Burkhardt J.K., Bingham J.B., Yu H., Olivo J.C., Schroer T.A., Hyman A.A., Griffiths G. (1997). Molecular requirements for bi-directional movement of phagosomes along microtubules. J. Cell Biol..

[bib6] Daitoku H., Isida J., Fujiwara K., Nakajima T., Fukamizu A. (2001). Dimerization of small GTPase Rab5. Int. J. Mol. Med..

[bib7] Dermine J.F., Scianimanico S., Privé C., Descoteaux A., Desjardins M. (2000). Leishmania promastigotes require lipophosphoglycan to actively modulate the fusion properties of phagosomes at an early step of phagocytosis. Cell. Microbiol..

[bib8] Dermine J.F., Duclos S., Garin J., St-Louis F., Rea S., Parton R.G., Desjardins M. (2001). Flotillin-1-enriched lipid raft domains accumulate on maturing phagosomes. J. Biol. Chem..

[bib9] Dermine J.F., Goyette G., Houde M., Turco S.J., Desjardins M. (2005). Leishmania donovani lipophosphoglycan disrupts phagosome microdomains in J774 macrophages. Cell. Microbiol..

[bib10] Desjardins M., Griffiths G. (2003). Phagocytosis: latex leads the way. Curr. Opin. Cell Biol..

[bib11] Desjardins M., Huber L.A., Parton R.G., Griffiths G. (1994). Biogenesis of phagolysosomes proceeds through a sequential series of interactions with the endocytic apparatus. J. Cell Biol..

[bib12] Erickson R.P., Jia Z., Gross S.P., Yu C.C. (2011). How molecular motors are arranged on a cargo is important for vesicular transport. PLoS Comput. Biol..

[bib13] Ghosh J., Bose M., Roy S., Bhattacharyya S.N. (2013). Leishmania donovani targets Dicer1 to downregulate miR-122, lower serum cholesterol, and facilitate murine liver infection. Cell Host Microbe.

[bib14] Gotthardt D., Dieckmann R., Blancheteau V., Kistler C., Reichardt F., Soldati T. (2006). Preparation of intact, highly purified phagosomes from Dictyostelium. Methods Mol. Biol..

[bib15] Goyette G., Boulais J., Carruthers N.J., Landry C.R., Jutras I., Duclos S., Dermine J.F., Michnick S.W., LaBoissière S., Lajoie G. (2012). Proteomic characterization of phagosomal membrane microdomains during phagolysosome biogenesis and evolution. Mol. Cell. Proteomics.

[bib16] Habermann A., Schroer T.A., Griffiths G., Burkhardt J.K. (2001). Immunolocalization of cytoplasmic dynein and dynactin subunits in cultured macrophages: enrichment on early endocytic organelles. J. Cell Sci..

[bib17] Harrison R.E., Bucci C., Vieira O.V., Schroer T.A., Grinstein S. (2003). Phagosomes fuse with late endosomes and/or lysosomes by extension of membrane protrusions along microtubules: role of Rab7 and RILP. Mol. Cell. Biol..

[bib18] Harrison R.E., Brumell J.H., Khandani A., Bucci C., Scott C.C., Jiang X., Finlay B.B., Grinstein S. (2004). Salmonella impairs RILP recruitment to Rab7 during maturation of invasion vacuoles. Mol. Biol. Cell.

[bib19] Hendricks A.G., Perlson E., Ross J.L., Schroeder H.W., Tokito M., Holzbaur E.L. (2010). Motor coordination via a tug-of-war mechanism drives bidirectional vesicle transport. Curr. Biol..

[bib20] Huynh K.K., Gershenzon E., Grinstein S. (2008). Cholesterol accumulation by macrophages impairs phagosome maturation. J. Biol. Chem..

[bib21] Ito J., Nagayasu Y., Yokoyama S. (2000). Cholesterol-sphingomyelin interaction in membrane and apolipoprotein-mediated cellular cholesterol efflux. J. Lipid Res..

[bib22] Johansson M., Rocha N., Zwart W., Jordens I., Janssen L., Kuijl C., Olkkonen V.M., Neefjes J. (2007). Activation of endosomal dynein motors by stepwise assembly of Rab7-RILP-p150Glued, ORP1L, and the receptor betalll spectrin. J. Cell Biol..

[bib23] Klopfenstein D.R., Tomishige M., Stuurman N., Vale R.D. (2002). Role of phosphatidylinositol(4,5)bisphosphate organization in membrane transport by the Unc104 kinesin motor. Cell.

[bib24] Lebrand C., Corti M., Goodson H., Cosson P., Cavalli V., Mayran N., Fauré J., Gruenberg J. (2002). Late endosome motility depends on lipids via the small GTPase Rab7. EMBO J..

[bib25] Mallik R., Carter B.C., Lex S.A., King S.J., Gross S.P. (2004). Cytoplasmic dynein functions as a gear in response to load. Nature.

[bib26] Mallik R., Petrov D., Lex S.A., King S.J., Gross S.P. (2005). Building complexity: an in vitro study of cytoplasmic dynein with in vivo implications. Curr. Biol..

[bib27] Mallik R., Rai A.K., Barak P., Rai A., Kunwar A. (2013). Teamwork in microtubule motors. Trends Cell Biol..

[bib28] Mayor S., Rao M. (2004). Rafts: scale-dependent, active lipid organization at the cell surface. Traffic.

[bib29] Noda Y., Okada Y., Saito N., Setou M., Xu Y., Zhang Z., Hirokawa N. (2001). KIFC3, a microtubule minus end-directed motor for the apical transport of annexin XIIIb-associated Triton-insoluble membranes. J. Cell Biol..

[bib30] Pollock N., de Hostos E.L., Turck C.W., Vale R.D. (1999). Reconstitution of membrane transport powered by a novel dimeric kinesin motor of the Unc104/KIF1A family purified from Dictyostelium. J. Cell Biol..

[bib31] Poser I., Sarov M., Hutchins J.R., Hériché J.K., Toyoda Y., Pozniakovsky A., Weigl D., Nitzsche A., Hegemann B., Bird A.W. (2008). BAC TransgeneOmics: a high-throughput method for exploration of protein function in mammals. Nat. Methods.

[bib32] Rai A.K., Rai A., Ramaiya A.J., Jha R., Mallik R. (2013). Molecular adaptations allow dynein to generate large collective forces inside cells. Cell.

[bib33] Rao M., Mayor S. (2014). Active organization of membrane constituents in living cells. Curr. Opin. Cell Biol..

[bib34] Rocha N., Kuijl C., van der Kant R., Janssen L., Houben D., Janssen H., Zwart W., Neefjes J. (2009). Cholesterol sensor ORP1L contacts the ER protein VAP to control Rab7-RILP-p150 Glued and late endosome positioning. J. Cell Biol..

[bib35] Simons K., Ikonen E. (1997). Functional rafts in cell membranes. Nature.

[bib36] Soppina V., Rai A., Mallik R. (2009). Simple non-fluorescent polarity labeling of microtubules for molecular motor assays. Biotechniques.

[bib37] Soppina V., Rai A.K., Ramaiya A.J., Barak P., Mallik R. (2009). Tug-of-war between dissimilar teams of microtubule motors regulates transport and fission of endosomes. Proc. Natl. Acad. Sci. USA.

[bib38] Soppina V., Norris S.R., Dizaji A.S., Kortus M., Veatch S., Peckham M., Verhey K.J. (2014). Dimerization of mammalian kinesin-3 motors results in superprocessive motion. Proc. Natl. Acad. Sci. USA.

[bib39] Sun J., Deghmane A.E., Soualhine H., Hong T., Bucci C., Solodkin A., Hmama Z. (2007). Mycobacterium bovis BCG disrupts the interaction of Rab7 with RILP contributing to inhibition of phagosome maturation. J. Leukoc. Biol..

[bib40] Turco S.J., Hull S.R., Orlandi P.A., Shepherd S.D., Homans S.W., Dwek R.A., Rademacher T.W. (1987). Structure of the major carbohydrate fragment of the Leishmania donovani lipophosphoglycan. Biochemistry.

[bib41] Vale R.D. (2003). The molecular motor toolbox for intracellular transport. Cell.

[bib42] Vallee R.B., McKenney R.J., Ori-McKenney K.M. (2012). Multiple modes of cytoplasmic dynein regulation. Nat. Cell Biol..

[bib43] Verhey K.J., Hammond J.W. (2009). Traffic control: regulation of kinesin motors. Nat. Rev. Mol. Cell Biol..

[bib44] Vieira O.V., Botelho R.J., Grinstein S. (2002). Phagosome maturation: aging gracefully. Biochem. J..

