# Supplementary material for: Dynein Clusters into Lipid Microdomains on Phagosomes to Drive Rapid Transport toward Lysosomes
Source: Cell. 2016 Feb 11;164(4):722–34. doi: 10.1016/j.cell.2015.12.054 (PMC4752818; doi:10.1016/j.cell.2015.12.054)
Supplement: Document S1. Supplemental Experimental Procedures [file mmc1.pdf]

Cell, Volume *164*

Supplemental Information

Dynein Clusters into Lipid Microdomains on Phagosomes to Drive Rapid Transport toward Lysosomes

Ashim Rai, Divya Pathak, Shreyasi Thakur, Shampa Singh, Alok Kumar Dubey, and Roop Mallik

SUPPLEMENTAL EXPERIMENTAL PROCEDURES AND REFERENCES

Dynein clusters into Lipid microdomains on phagosomes ... (Ashim Rai et al)

SUPPLEMENTAL EXPERIMENTAL PROCEDURES

1) Materials

All materials were purchased from Sigma-Aldrich, Bengaluru, India unless otherwise mentioned. Carboxylated latex beads (759 ± 23 nm diameter) were from Polysciences (Warrington). Silica beads (1 µm and 2 µm diameter) were from Corpuscular Inc. (Cold Spring, NY). Phospholipids used for TLC were from Avanti Polar Lipids, Inc. (Alabaster, Alabama). Rab7 antibody (Cat. # 9367) was from Cell Signalling Technology (Danvers, MA). Flotillin-1 (Cat. # 610821) and dynactin p50 (Cat. # 611002) antibodies were from BD Biosciences (San Jose, CA). Actin (Cat. # AAN01-A) antibody was from Cytoskeleton, Inc. (Denver,CO). Dynein intermediate chain antibody IC 74-1 (Cat. # sc-13524) was from Santa Cruz Biotechnology (Dallas,Texas). Antibody against *Dictyostelium* dynein heavy chain was a kind gift from M. Koonce. HL-5 medium (Cat. # HLC0102) used for *Dictyostelium* cell culture was from Formedium, UK. Alexa Fluor 594 conjugated Cholera Toxin Subunit B (Cat.# C-22842) was from Life Technologies, Bengaluru, India. Antibody against Golph3 was from Sigma-Aldrich (SAB4200341).

2) Cell Culture

Dictyostelium cells of AX-2 strain (acquired from Dictybase) were grown in HL-5 medium at 22˚C in suspension culture (Barak et al., 2014). J774.2 macrophage cells were cultured as previously described (Rai et al., 2013).

3) Microscope Setup and Data Acquisition

The instrument and detection system have been previously described (Barak et al., 2013) and used by us to assay motion of *Dictyostelium* endosomes in cell extracts (Soppina et al., 2009b) and latex bead phagosomes inside J774 cells (Rai et al., 2013). Motion was observed using DIC microscopy (Nikon, Melville, NY, USA) with a 100X, 1.4 NA oil objective. Image acquisition was done at video rates (30 frames/s, no binning) with a Cohu 4910 camera. Position tracking of phagosome motion was done offline with custom written software in Labview (National Instruments) using a tracking algorithm which calculates position of the centroid of a cross-correlation image with sub-pixel resolution (Carter B.C. et al., 2005).

4) Optical trapping and Quadrant photodiode detection

The optical trapping setup has been described (Barak et al., 2013). A single mode diode laser at 980nm (Axcel photonics) was used after beam expansion to fill the back focal plane of the objective. The laser power at the sample plane was between 20-70 mW, which has been shown to cause minimal optical damage to the specimens (Mallik R. et al., 2004). A quadrant photodiode (QPD) was used to obtain stall force records and thermal fluctuation data for measuring trap stiffness. Stall force data was digitized at 2kHz. Thermal fluctuations were recorded at 40kHz. For measuring trap stiffness of phagosomes, the video-matching method (V_{MATCH}) was used (Barak et al., 2013). For 759 nm phagosomes, the trap stiffness was in the range of 0.1 - 0.13 pN/nM. For stall force measurements, 150 nm displacements from the trap centre were chosen as the "cut-off" for the linear range of the optical trap. The linear range of both the QPD and optical trap had been measured separately. The chosen cut-off for measuring stalls falls within the measured range.

5) Agar overlay method to assay in vivo motility of phagosomes in *Dictyostelium* cells

The agar-overlay technique was adapted from Fukui Y. et al (Fukui et al., 1987). $2x10^7$ cells/mL of *Dictyostelium discoideum* AX2 strain were pulsed with 759nm latex beads at a final density of ~$1x10^8$ particles/mL for 5min followed by a chase in Na/K-phosphate buffer (pH 6.5) for 40 min. Approximately 40µl of this suspension was placed on a glass slide and the cells were allowed to adhere for 5 min. A 7x7mm square wide 2% agarose sheet (prepared in Na/K-phosphate buffer pH 6.5) was placed on the coverslip and excess liquid was blotted off using a tissue paper. The cells were then imaged with a 100X oil objective (1.4 NA) under a DIC microscope.

6) Preparation, Purity and *In vitro* motility of Latex bead phagosomes from *Dictyostelium*

<u>PREPARATION</u>

The in vitro reconstitution of phagosome motility in cell extracts has been described previously (Barak et al., 2014). Briefly, latex beads (759 nm diameter, Polysciences; $3.5x10^{10}$ beads) were washed twice with HL-5 medium by centrifugation (11000*g* for 5 min at 4°C). The beads were resuspended in 0.5 mL Sorensen's

buffer, sonicated for 10 min on a bath sonicator and kept on ice. AX-2 *Dictyostelium* cells were grown in suspension cultures at 22°C to a cell density of 4-6x10^6cells/mL. Cells (4-6x10^8) were collected by centrifugation at 900*g* for 5 min at room temperature. The cell pellet was stored on ice and resuspended in 5mL of Sorensen's buffer. Beads were added to the cell suspension and incubated for 15min at 4°C with gentle mixing to ensure synchronous uptake of beads. For starting the pulse, the bead-cell mixture was added to 100mL of HL-5 medium kept at 22°C and shaken at 150rpm to initiate a pulse-chase routine for phagosome maturation. For preparing early phagosomes, the cells were pulsed with beads for 5min with no chase. For late phagosomes, cells were pulsed with beads for 15min and chased for 45min. To stop pulse/chase, the cells in suspension culture were immediately added to 330 mL of ice-cold Sorensen sorbitol buffer (SSB) and centrifuged at 900*g* for 5min at 4°C. To remove non-phagocytosed beads, the cell pellet post-chase was washed twice with Sorensen's buffer by centrifuging at 900*g* for 5min at 4°C. The cell pellet was finally resuspended in 1:1 (w/v) of lysis buffer containing 30% sucrose (LB-30: 30mM Tris, 4mM EGTA, Roche protease inhibitor cocktail, complete EDTA-free at 2X concentration, 3 mM DTT, 20 µg/mL Pepstatin A, 5 mM PMSF [phenyl methyl sulfonlyl fluoride], 5 mM Benzamidine hydrochloride containing 30% sucrose). The cells were lysed by passing once through a 5µm polycarbonate filter. The cell lysate was layered over a 1mL cushion of LB-25 (LB containing 25% sucrose) and centrifuged in MLS-50 rotor (Beckman) at 100000*g* for 20min at 4°C. The phagosome-extract mixture was collected from top of the LB-25 cushion and snap frozen as 40µL aliquots in liquid nitrogen for later use in motility assays.

PURITY

A phagosome prep was done along with a sham prep (no beads added) using the same number of *Dictyostelium* cells. Both samples were subjected to sucrose gradient centrifugation (Fig S2-A). The 10-25% sucrose interface had a milky appearance for the phagosome prep (due to scattering of light from phagosomes). A clear solution devoid of any organelles/membranes was seen at the 10-25% interface in the sham prep. This 10-25% interface solution was subjected to SDS-PAGE gel, where no protein could be detected for the sham prep, but abundant proteins were seen in the latex bead phagosome prep (Fig S2-A). Therefore, no detectable proteins/membranes can float into the 10-25% sucrose interface in the absence of phagocytosed beads.

The identity of Early and Late phagosomes was validated using immuno fluorescence staining of EPs and LPs purified from J774 cells. Antibody against EEA1 (Early endosome antigen 1; early endosome marker) was used. Antibody against LAMP1 (Late endosome/phagosome marker) were used. EEA1 and LAMP1 were enriched respectively on EPs and LPs (Fig S2-B and Fig S2-C). These results show that the pulse-chase strategy yields phagosomes at a defined stage of maturation (early or late). This is also confirmed by Western blotting of purified EPs and LPs against known phagosomal markers (Fig S2-D).

Possibility of contamination of the latex bead phagosome fraction with cytosolic and/or membrane from other organelles was investigated using antibodies against a range of organellar marker proteins:- Cytosol (tubulin), Plasma membrane marker (Na,K-ATPase), Endoplasmic Reticulum markers (KDEL and PDI), Mitochondrial marker (Hsp60), and Golgi marker (GOLPH3). Latex bead phagosomes were purified from RAW264.7 mouse macrophage cells. Cell lysate was also prepared from the same cells. Equal amount of protein from phagosomes and cell lysate was loaded in an SDS PAGE gel to separate the proteins. Western blotting was then performed using antibodies to the marker proteins mentioned above. All these marker proteins could be detected in the cell lysate, but were not detected in the phagosome fraction (Fig S2-E). This suggests that latex bead phagosomes are largely free of membrane and cytoplasmic contamination. Two bands for the KDEL marker indicate two ER proteins GRP78 and GRP94 which have the KDEL sequence.

IN VITRO MOTILITY ASSAY

The motility assay with phagosomes in cell extracts has been described (Barak et al., 2014). Briefly, a motility mixture was prepared by adding a 20x ATP regenerating mix (ATR = 20mM ATP, 20mM $MgCl_2$, 40mM sodium creatine phosphate and 40U/mL creatine phosphokinase). This mixed with a 40µL aliquot of phagosome extract to obtain a final ATP concentration of 1mM ATP during the motility assay. The motility mixture was then passed through a flow chamber containing *in vitro* polymerized microtubules which had been polarity marked at their minus ends as described (Soppina et al., 2009a). Robust motility could be observed for upto 20min at 22°C.

7) Purification of Silica bead phagosomes from J774 and RAW264.7 cells cells and fluorescence staining of phagosomes.

Five culture dishes (10cm diameter) plated with J774 or RAW264.7 mouse macrophage cells at 70% confluence were used for phagosome preparation. 500µl of 2µm diameter silica bead stock was washed

thrice with incomplete DMEM (10,000*g,* 5min, 4°C) and resuspended in 1ml of incomplete DMEM. The bead solution was then briefly vortexed, sonicated for 2mins and added to 15ml of pre-warmed (37°C) incomplete DMEM and mixed well. 3ml of the bead suspension was then added to each of the 10cm dishes. An initial pulse of 30min and 10min duration was given for preparation of late and early phagosome respectively. The incomplete DMEM was then exchanged with complete DMEM and a chase of 5hrs and 10min was given for late and early phagosome respectively. The pulse and chase were done at 37°C. The dishes were then given three washes with 5ml of ice cold PBS each and then gently scraped off with 5ml of PBS per dish. All the steps thereafter were done in a cold room. The cells were pelleted at 3000rpm, 4°C for 5mins in a 50ml falcon (swinging bucket rotor, Eppendorf) and resuspended in 25ml of homogenisation buffer (250mM sucrose, 3mM Imidazole, pH 7.4). The centrifugation step was repeated and the pellet was resuspended in 1ml of Lysis buffer [Homogenisation buffer, 3mM DTT (Sigma), 5mM Benzamidine (Sigma), 5mM PMSF (phenylmethyl-sulfonyl fluoride, Sigma), PI Cocktail (Roche), Pepstatin A (Sigma)]. Lysis was carried out in a 1ml dounce (Wheaton), until 90% of lysis has been achieved (sample was checked in between to quantify lysed percentage). The lysed cell sample was overlayed on a sucrose step gradient made by 5ml (each) of 85% and 65% sucrose solution, containing 3mM Imidazole and protease inhibitors of same concentration as mentioned before (14x89 mm centrifuge tubes, Beckman). The gradient was centrifuged in a Beckman SW41 rotor at 100000*g* for 1hour. Purified phagosomes were collected at the bottom of the tube, along with the un-lysed cell debris pellet, forming a fine layer over it. The sucrose solutions were discarded and the centrifuge tube was cut (keeping 10 cm from the bottom) with a blade. Using 200µl of TNE buffer (50mM Tris pH 7.4, 140mM NaCl, 5mM EDTA) containing the above mentioned protease inhibitors, the fine layer of purified phagosomes were gently pipetted in and out 4-5 times and collected. The sample was then aliquoted (20µl each), snap frozen and stored in liquid nitrogen. The phagosomes were stable for a week.

Acid washed coverslips were incubated in poly-L-lysine solution containing 18 ml Ethanol (AR, 99.9%), 2ml Methanol (AR, 99.9%) and 1 ml poly-L-Lysine (Sigma, 0.1% w/v in H_2O) for 20 min at RT. The coverslips were then dried in a preheated oven (100°C) for 10min. The coverslips were allowed to cool down, and then placed in a 24 well plate. Pre-aliquoted purified phagosomes (20µl) were used per coverslip (per well) and centrifuged at 1200*g* for 5 min using multi-plate well swinging bucket rotor (Eppendorf). The coverslips with phagosomes stuck on them were washed once with PBS and then fixed with 4% PFA for 15min, followed by three washes with PBS and treatment of 200mM Glycine in PBS to quench the free aldehyde groups. Phagosomes were treated with 30mM Methyl-β-cyclodextrin (Sigma) for 5minutes before proceeding to fixing and Glycine treatment steps in experiments designed to probe the effect of cholesterol sequestering. Next, phagosomes were incubated for 1hour with 6% BSA (Sigma) in PBS and then treated with the primary antibodies for 1 hour at room temperature or overnight at 4°C. Rabbit polyclonal Anti-Flotillin 1 antibody was used at 1:50 dilution and co-immunostained with mouse monoclonal anti-Dynein antibody (Intermediate Chain, D5167 SIGMA), also used at 1:50 dilution. Only dynein immunostaining was done at 1:25 dilution in all other cases. Rabbit polyclonal anti-Lamp 1 antibody (ab24170) and Rabbit polyclonal anti-Rab7 antibody (Cell signalling, 2094S) were used at 1:100 dilution. For visualization Donkey anti-Rabbit Alexa fluor 488 and Donkey anti-Mouse Alexa fluor 555 secondary antibodies were used (Molecular probes). Vectashield (Vectorlabs) was used as the mounting media to reduce photo bleaching.

Imaging was done using a Zeiss LSM 510 Confocal microscope. The images to investigate colocalization of dynein and Rab7 were taken by exciting the fluorophores in a sequential manner. To check for any possible cross talk, we excited a phagosome sample immunostained only for Dynein (Alexa 555) with the 488 nm laser, and acquired emission signal in the green channel. No signal could be observed under these conditions. Similarly, a phagosome sample immunostained for only Rab7/Flotillin (Alexa 488) was excited with 563 nm laser and signal was acquired in the red channel. Again, we did not observe any signal.

Filipin, an antibiotic that binds to cholesterol was used to detect cholesterol in lipid membranes (Maxfield and Wustner, 2012). Filipin was used at a final concentration of 0.083µg/µl in PBS. Phagosomes stuck on polylysine coated coverslip were incubated with filipin for 1hr at 22°C. The coverslips were then washed twice with PBS before mounting in Vectashield for imaging on a Nikon Eclipse Ti microscope. For immunostaining of phagosomes inside J774 cells, the cells were plated on coverslips and pulsed-chased as explained above using silica beads, when 70% confluent. Immunostaining protocol was same as above. For antibody labelling, the cells were fixed in 4% PFA for 5mins followed by washing and permeabilization using 0.1% Saponin for 1hour. The LAMP1 antibody was used at 1:100 dilution. The Alexa 488 goat anti-rabbit secondary antibody was used at 1:200 dilution. Imaging was done using a Zeiss LSM 510 Confocal microscope.

8) Measurement of fluorescence intensity along circumference of Phagosomes

The "segmented line" tool in ImageJ was used to carefully trace a circle along the fluorescent circumference of an EP or LP. Once the circle was complete, the "Plot profile" tool was used to obtain an array of numbers that is essentially the pixel intensity along the phagosome surface in that confocal section. This array is plotted as a function of angular rotation (θ) in Fig 3B for an EP and an LP. Similar plots are also shown for treated and MβCD/LPG treated samples in Fig S4 (G,H,I). All analysis was done in a blind manner (with no knowledge of the treatment condition of sample). We believe that this procedure can reliably estimate relative changes in protein amount on the phagosomes. This is because the fluorescence intensity results were in good agreement with changes in protein amount, as determined from Western blotting experiments on purified phagosomes (see main text).

9) Purification of Silica bead phagosomes from *Dictyostelium* and Immunofluorescence of purified phagosomes.

One ml of 2μm silica bead stock was washed thrice with 1mL HL-5 medium at 4˚C, resuspended in 500μl Sorensen's Buffer (SB), briefly sonicated to prevent clumping and kept on ice. *Dictyostelium* cells at cell density of 4-6×10^6 cells/ml from 100ml suspension culture were harvested by centrifugation at 900*g* and 4°C for 5min. Cells were washed twice with SB and finally resuspended them in 5ml of SB. Freshly prepared beads were added to the cell suspension and incubated on a rotator with gentle mixing at 4°C for 15min. Bead-cell suspension was poured into 100ml of HL-5 medium at 22°C to start 'pulse', for 5 minutes (for early phagosome) and 15 minutes (for late phagosome). The pulse was done in a shaking incubator (≈150 rpm). To stop the pulse cell suspension was transferred to 400ml ice-cold SB. Cells are harvested at 900*g* and 4°C for 5min and washed twice with 50ml ice cold SB. For early phagosomes, cells are lysed after this step. For late phagosome preparation, cells are resuspended in 5ml ice cold HL-5 medium and poured into 100ml of HL-5 medium at 22°C, followed by a chase of 45minutes. Ice cold SB is added to stop the chase and cells are harvested as stated above for lysis. Thereafter, all the steps are same for early and late phagosomes. For lysis cells were resuspended in LB/30% sucrose in 1:1 (w/v) ratio and passed through a syringe filter membrane (pore-size 5μm). Cell lysate was centrifuged in a sucrose density gradient (from bottom:- LB/80%, LB/65% and cell lysate) at 100000*g* at 4°C for 1hour. Silica phagosomes were collected (as a white layer) from the pellet at the bottom of the tube, resuspended in LB and aliquots were frozen in liq. N2.

Aliquots of 40μL purified phagosomes were diluted to 100μL in LB, then spread and adhered to the surface of poly-L-lysine coated coverslip by incubating for 5-7 minutes. The samples were washed thrice with PBS and fixed using 2% formaldehyde in methanol for 5 minutes at -20°C. Immunostaining was done using anti-dynein Heavy Chain (DHC) polyclonal rabbit antibody generously gifted by Dr Michael Koonce. A stock concentration of 1.4 mg/μl was used at 1:200 dilution in 1xPBS at 40ºC overnight. After treating the samples with 1:50 dilutuion of Alexa 555 for an hour the samples were washed thrice with PBS, coated with vectashield and imaged using the Zeiss LSM 510 confocal microscope.

10) Image analysis of Western blots/TLCs for estimating relative change in protein and lipid amount

We estimated the relative change in protein amount using ImageJ. The integrated intensity within a rectangular box enclosing the protein band was calculated for each band (e.g. see EP and LP bands for dynein in Fig S2-F (inset). A box of the same area was used for both bands. The same box was then moved to a region not containing any bands and integrated intensity within box calculated again to obtain the background intensity. This background was subtracted from the intensity of EP and LP dynein bands. Thereafter, the ratio of background-corrected EP:LP intensities was calculated. This ratio was taken as an estimate of the relative change in dynein levels between EPs and LPs. A similar method was used on dynein bands in Fig S4-E to estimate fold changes upon MBCD treatment (quantification shown in Fig S4-F).

For determining the relative change in PC between EPs and LPs we first prepared varying (known) amounts of pure PC samples by serial dilution. A Thin Layer Chromatograph (TLC) was run on this dilution series along with the EP and LP samples. The results were analysed by ImageJ (as described above for proteins) to obtain a calibration curve of PC (Inset, Fig S5-B). This calibration curve was used to obtain the amount of PC in EP and LP samples. PC constitutes 50% of the lipid content of latex bead phagosomes, and this number is remarkably conserved between EPs and LPs (Schroeder, 1982). We therefore assumed that equal number of EPs and LPs should have equal amount of PC (see main text).

11) Isolation of detergent-resistant membrane (DRM) fraction from phagosomes

The procedure used for DRM isolation from J774 cells was adapted from a protocol described previously (Goyette et al., 2012). J774 cells were cultured in Dulbecco's modified Eagle's medium high glucose

(Sigma) supplemented with 10% heat-inactivated fetal bovine serum, 1% glutamine at 37°C in a 5% CO_2 incubator. Cells were grown to 80% confluence in petri dishes prior to the experiment. Approximately $1.4x10^8$ cells were used for each experiment. Latex beads (759 nm diameter, Polysciences) were diluted 1:50 in culture medium without serum. J774 cells were pulsed with beads for 15min. After the pulse, cells were washed thrice with ice-cold phosphate-buffered saline (PBS) to remove unphagocytosed beads. Complete culture medium (with serum) was added to the cells and the cells were chased for 4 hours at 37°C to allow phagosomes to mature into late phagosomes. After the chase, the washing with ice-cold PBS was repeated as described above. The cells were then scraped with a rubber policeman using ice-cold PBS and collected. Cells were then pelleted at 1000*g* for 5min at 4°C. The cells were then washed once with homogenization buffer (HB: 250 mM sucrose, 3 mM Imidazole pH 7.4) by centrifuging at 1000*g* for 5min at 4°C. The cell pellet was stored on ice and resuspended in 1mL of HB containing protease inhibitors (Roche protease inhibitor cocktail, complete EDTA free Cat.# 11873580001 at 2x concentration, 10µg/mL Pepstatin A and 3mM DTT). Cells were lysed by homogenizing on ice using a Dounce homogenizer with a tight-fitting pestle. Lysis was continued until ~90% of cells had been lysed as confirmed microscopically using bright field microscopy. Unbroken cells were separated from the cell lysate by centrifuging at 1200 rpm for 5 min at 4°C. The supernatant was collected and mixed with an equal volume of 85% sucrose (in 3mM imidazole pH 7.4) to obtain a mixture with a ~40-45% final sucrose concentration.

A sucrose gradient was next set up as follows:- The cell lysate-sucrose mix was overlaid over a bottom layer of 1mL 62% sucrose (all sucrose solutions in 3mM imidazole pH7.4) followed by 2mL of 35% sucrose, 2mL of 25% sucrose and 2mL of 10% sucrose. The gradient was centrifuged in a SW41 swinging bucket rotor (Beckman) for 2 hrs at 100000 g at 4°C. The phagosomes were collected from the interface of 10-25% sucrose. Phagosomes were diluted 1:10 in PBS and centrifuged at 100000*g* for 1 hr at 4°C to pellet the phagosomes. The phagosome pellet was resuspended in 150µL of TNE buffer (25mM Tris, 150mM NaCl , 5mM EDTA) and kept on ice. The isolated phagosomes were brought to a final concentration of 1% Triton X-100 by adding an equal volume of 2% Triton X-100 in TNE buffer. The mixture was shaken gently for 30min at 4°C to solubilize phagosomal membranes. The solubilized membranes were collected by centrifuging the mixture at 15000 rpm for 5 min at 4°C. This centrifugation was repeated again after resuspending the phagosomes in TNE buffer to maximize membrane yield.

The gradient for DRM isolation was set up as follows: 0.6ml of 60% Optiprep™ stock in TNE buffer was added to the membrane fraction to obtain a final concentration of 40% Optiprep™. This mixture was added to the bottom of a MLS-50 rotor tube followed by 3 ml of 30% Optiprep™ stock in TNE buffer and overlaid with 0.6 mL of TNE buffer. The DRM gradient was centrifuged at 165000g for 4 hrs at 4°C in Beckman MLS-50 swinging bucket rotor on a table top ultracentrifuge. Equal volume samples of 0.6mL were collected from the top of the gradient and were numbered accordingly with the topmost fraction being fraction 1. Fraction 1 & 2 which are known to be DRM fractions (Goyette et al., 2012) were pooled and processed further for protein precipitation using methanol-chloroform method as described elsewhere (Goyette et al., 2012). Similarly, fractions 7 & 8 which are the detergent soluble fractions (DSFs) were pooled and processed as above for sample preparation.

12) Measurement of Cholesterol on phagosomes by Cholesterol assay kit, LC/MS/MS, direct infusion MS/MS and GC-MS

Phagosomes were prepared using silica beads as described earlier. Lipids were extracted according to the method of Bligh and Dyer (Bligh and Dyer, 1959). Briefly, 0.8ml aqueous sample containing silica bead phagosomes was mixed with 2ml of Methanol and 1ml chloroform followed by vortexing and overnight lipid extraction. 1ml each of chloroform and water were added the next day which resulted in phase separation; the lower being the organic phase. The organic phase was transferred to a new glass tube, dried under a stream of N_2 and resuspended in 10-15µl of chloroform. The silica TLC plates (Merck) were pre-cleaned using Chloroform followed by air drying and heating to 100˚C for 15 mins. The sample was then spotted onto these plates using a glass capillary. The solvent system used was that of Downie and Kealey with minor modifications (Downie and Kealey, 1998). The first solvent was a mixture of ethyl acetate: Isopropanol: Chloroform: Methanol: 0.25% KCl (25:25:25:10:9 v/v). The first solvent was run halfway upto the top of the plate, after which the the plate was air dried. The plate was then run in two times in neutral solvents. The first being Toluene: Diethyl ether: ethanol: acetic acid (60:40:1:0.23 v/v) after which the plate was dried and ran in hexane: diethyl ether (94:6). The plate was dried and visualized by spraying with 10% $CuSO_4$ in 8% H_3PO_4 followed by baking in the oven above 150˚C for 15-20 minutes. Image of the plate was taken on the BioRad instrument using white light illumination.

Comparison of cholesterol first required us to prepare an EP and an LP sample with the same total membrane area. Phagosomes prepared with beads offer the unique advantage that an EP has exactly the same spherical shape and size as an LP. This is the size of enclosed bead if the thickness of tightly apposed membrane (~5nm) is ignored. We assume that the bilayer membrane is tightly apposed to the spherical bead for all phagosomes, as indeed seen from the fluorescent staining of membrane-bound proteins on EPs and LPs (Figs 3A, 3F, 3G and 3H). Therefore, an EP sample and an LP sample with the same number of phagosomes should have the same total surface area, and therefore the same amount of total membrane lipids. In order to obtain samples with equal number of EPs and LPs, we adjusted the EP and LP samples by dilution to yield the same value of optical density (OD) at 600nm (= OD_{600}). This method has been used extensively to normalize latex bead phagosome samples (Blocker et al., 1997; Blocker et al., 1996; Desjardins et al., 1994; Gotthardt et al., 2002). The method was tested with a series of samples containing known amount of beads, and yielded a linear variation in OD at 600nm (Fig S5-A; also see Methods). Having thus obtained EP and LP samples with approximately equal number of phagosomes, we measured the amount of phosphatidylcholine (PC) in these samples using thin layer chromatography (TLC). PC, being an essential phospholipid, constitutes 50% of the lipid content of latex bead phagosome membranes. The amount of PC is remarkably conserved between EPs and LPs (Schroeder, 1982). We therefore used the levels of PC to confirm that EP and LP samples had an equal number of phagosomes. Fig S5-B (inset) shows the bands for PC in a TLC run for an EP and an LP sample having equal OD_{600}. A calibration curve for the TLC band intensity (using known amounts of PC) is also shown. The amount of PC in EP and LP samples, as calculated from this curve is also shown. The EP sample showed slightly higher PC (~8%), suggesting that the method of normalizing samples by OD_{600} works well.

To further confirm the relative levels of PC, we measured the amount of total PC in EP and LP samples using direct infusion mass spectrometric analysis of PC (MS/MS). This experiment was done at the lipidomics facility, National Centre for Biological Sciences (NCBS), Bangalore India. Mass spectrometric analyses was performed on a high resolution mass spectrometer; LTQ Orbitrap XL (Thermo Fisher Scientific, Bremen, Germany). The sample was directly infused in to the mass spectrometer using a robotic nanoflow ion source TriVersa NanoMate (Advion BioSciences, Ithaca, NY). The ion source is equipped with the chips with the diameter of spraying nozzles of 4.1 mm. It was controlled by Chipsoft 8.3.1 software. Ionization voltages were 1.2 kV in positive mode. The temperature of ion transfer capillary was 180°C; tube voltage was 40 V (MS-). All acquisitions were performed at the mass resolution $R_{m/z\ 400}$ = 100 000. In full scan mode mass range was m/z 350 to 1200. AGC control was set at $2x10^6$ ions and maximum injection time was set to 500 ms. Dried total lipid extracts were re-dissolved in 100 ml of chloroform:methanol 1:2. For the analysis, 50 µl of samples were loaded onto 96-well plate (Eppendorf, Hamburg) of the TriVersa NanoMate ion source. Before loading, the plate was sealed with aluminum foil. Each sample was analyzed for 5 min in negative ion mode for detection and quantification of PC. Acquisition delay time was 20 sec to stabilize spray current. MS/MS fragmentation was carried out in FT HCD mode using Orbitrap. Mass resolution was maintained at $R_{m/z\ 400}$ = 30000 for fragmentation throughout the run. Normalized collision energy was maintained between 25 to 28 V. The EP sample was again found to have slightly higher PC (~8%) than the LP sample by mass spectrometry (Fig S5-C). We therefore adjusted the EP and LP samples by dilution to more precisely match the PC levels, and then used these "PC-equalized" samples for cholesterol measurement.

We used a commercially available colorimetric assay kit (Cholesterol assay Kit from Abcam ab65390) to measure free cholesterol on EP and LP samples having equal PC (Li et al., 2011). The manufacturer-provided protocol for colorimetric assay was followed. In this assay, cholesterol oxidase recognizes free cholesterol and produces products that generate colour. The amount of PC on LPs was determined to be 1.1 times that of EPs from image analysis of the TLCs (Fig S5-B) and LC-MS (Fig S5-C). Therefore, LPs were re-suspended in 796µl and EPs in 700µl of cholesterol assay buffer to adjust for this difference. Samples were incubated at 37ºC for 1 hour before measurement of optical density (OD) at 570nm (OD_{570}) in a 96-well plate. Known cholesterol standards were used to prepare a calibration curve that showed linear variation in OD_{570} with cholesterol amount. Fig S6-A and Fig S6-B show the results of this colorimetric assay. Note that the measured cholesterol on EPs and LPs (red dots) falls within the linear dynamic range of this experiment. By this assay, we measured 1.79 fold more cholesterol on LPs compared to EPs (Fig S6-B).

We employed a targeted quantitative lipidomics approach to measure the cholesterol in phagosome membranes as a function of maturation. We subjected the lipids extracted from EP and LP samples to liquid chromatography - mass spectrometry (LC/MS/MS). These experiments to measure PC and cholesterol were done by Avanti Polar Lipids (Alabaster, Alabama). Dried lipid samples were obtained from purified EPs and LPs that were prepared by phagocytosis into RAW264.7 cells. These samples were

shipped to Avanti Polar under argon. Samples were dissolved directly with 1ml of 1:1 chloroform:methanol. These solutions were initially subjected to LC/MS/MS to estimate the need for dilution. A 1:100 dilution was done into an internal standard solution containing 17:1 lyso PC and 17:0-20:4 PC. The PC molecular species were semi-quantified as the area ratio of analyte/ISTD multiplied by ISTD concentration. The cholesterol was measured by LC/MS/MS directly from the original 1ml solution against a calibration curve of cholesterol reference material. The amount of cholesterol in both samples (after normalizing to PC) is plotted in Fig S6-C. We observed 1.35 fold more cholesterol in LP versus EP samples.

We subjected lipids extracted from the EP and LP samples to gas chromatography - mass spectrometry (GC-MS) for measurement of Cholesterol. These experiments were done at the Centre for Cellular and Molecular Platforms (C-CAMP), Bangalore, India. GC-MS was performed with a Perkin Elmer gas chromatograph (Clarus SQ 8C) interfaced with a single quadrupole. The electron energy was 70eV and the ion source temperature was 230°C. Each sample (1 µl) was injected in Splitless mode at 250°C and separated through a RTX-5MS cross bond 5% diphenyl / 95% dimethylpolysiloxane capillary column (30m x 0.32mm inner diameter, 0.25 µm film thickness, Restek). The oven temperature was held initially at 150°C for 1 min, ramped to 290°C at 7°C/min (held for 3 min), increased to 300°C at 10°C/min . The carrier gas was ultra-high purity helium at a column head pressure of 1034.21 kPa (150 Psi; column flow 1 ml/min at oven temperature 300°C). Pure cholesterol standards (Sigma Aldrich) were used at different concentrations to plot a standard curve and determine the linear range of detection for the instrument. The cholesterol standards gave a single peak at RT= 19.36 and similar peak in the phagosome sample was used for cholesterol quantification. The samples were run in duplicates and also in different dilutions for GC-MS analysis. Known cholesterol standards were used to calibrate the instrument, and yielded a prominent peak for cholesterol at retention time (RT) = 19.36sec. A peak was also detected for EP and LP samples at RT=19.36 sec, and was therefore ascribed as the peak for cholesterol (Fig S6-D). The area under this peak reflects the abundance of cholesterol in a sample (values mentioned in Fig S6-D). Fig S6-E shows a calibration curve of known cholesterol standards using GC-MS. A linear variation in cholesterol abundance is seen over this range of cholesterol standards, thus verifying the dynamic range of GC-MS measurements. The abundance of cholesterol for EP and LP samples is also shown in Fig S6-E (red dots), and is within the linear range of cholesterol standards. This calibration curve yields 1.85-fold higher cholesterol on LPs as compared to EPs (see inset of Fig S6-E).

13) Cyclodextrin and Lipophosphoglycans treatment of phagosomes for imaging, Western blotting and motility assay

Methyl β-cyclodextrin (MβCD) prepared in LB buffer (30 mM Tris, 4 mM EGTA ,pH 8.0) was added to the phagosome extracts at final concentrations ranging from 10mM to 30mM MβCD. After addition of MβCD, the mixture was incubated at 22°C for 15 min. For mock buffer treated samples, an equal volume of LB buffer was added to the phagosome extracts and the incubation was done exactly as described above. ATP was added to the mixture and phagosome motility was assayed as described above. Such identically treated samples were used for imaging, Western blotting and motility assays.

LPG purified from *Leishmania donovani* (Turco et al., 1987) was obtained as a gift. A stock solution of LPG (0.5mg/ml) in distilled water was diluted appropriately for motility and imaging experiments. LPs were incubated with LPG (22°C, 15 min) before observation, as reported elsewhere (Dermine et al., 2005). The molecular weight of LPG was 9kDa (personal communication from S.J. Turco). The final LPG concentrations used in experiments were 12.5µg/ml, 37.5µg/ml and 62.5µg/ml. The molar concentrations of LPG used were calculated to be 1.4µM, 4.2µM and 6.9µM.

14) Preparation of ATP releasate from *Dictyostelium* cells and bead motility.

The protocol for preparation of ATP releasate has been adapted from earlier work (Pollock N. et al., 1998) but is briefly described here. *Dictyostelium* cells (1 litre) were grown to a cell density of 4-8x10^6 cells/mL at 22˚C in suspension culture. The cells were pelleted by centrifuging at 900*g* for 5min at 4˚C. The cells were washed twice in 500mL of ice-cold Sorensen's buffer and the cell pellet was stored on ice. The cell pellet was resuspended in a 1:1 (w/v) ratio in LB-30 and the cells were lysed by one passage through a 5µm polycarbonate filter. The cell lysate was collected and spun at 1400*g* for 5min at 4˚C to prepare a post-nuclear supernatant (PNS). The PNS was overlaid over a cushion of LB-25 and centrifuged at 180000*g* for 15min at 4˚C in Beckman MLS-50 rotor to prepare a high-speed supernatant (HSS). Prior to HSS preparation, 500µl of goat brain tubulin (30 mg/mL) was polymerized with 10% DMSO and 1mM GTP in 40mM K-Pipes, pH 6.8, 0.5mM EGTA, 2mM $MgCl_2$ (0.5x BRB80) and 20µM taxol by incubation for 45 min at 37°C. The microtubules were collected by centrifugation at 360,000*g* for 10min in a Beckman TLA100 rotor (22°C). The HSS was incubated with 15 U/ml hexokinase, 3 mM glucose, 4mM AMP-PNP/$MgCl_2$, and

20µM taxol. The microtubule pellet was resuspended in HSS to a final tubulin concentration of 0.5 mg/ml and incubated on ice for 20 min. The microtubule and associated proteins were then centrifuged through a 1mL cushion of LB-25 containing 20µM taxol at 85000*g* for 15min at 4˚C in a Beckman MLS-50 rotor. The microtubule pellet was resuspended in LB-5% sucrose containing 5mM ATP/$MgCl_2$ (1:20 of original HSS volume) to release the motor proteins. Microtubules were separated from the ATP-released proteins by centrifuging at 90000*g* for 15min at 4˚C in Beckman MLA-130 rotor. The resulting supernatant was the ATP releasate, which was snap frozen immediately in liquid nitrogen in 25µl aliquots and used later for assaying motility.

Carboxylated latex beads (759nm diameter) were washed twice with LB buffer. The beads were finally resuspended in LB buffer at 1:50 dilution of the original bead stock, sonicated and stored on ice till further use. A 20x ATP regenerating mix (20mM ATP, 20mM $MgCl_2$, 40mM creatine phosphate, and 40 U/ml creatine kinase) was prepared and stored on ice. The motility mixture was prepared by mixing 25µl of ATP releasate, 20µl of bead solution and 5µl of 20x ATP regenerating mix. The motility mixture was incubated for 10 min on ice and then directly assayed for motility in a flowcell. For MβCD treatment, the motility mixture was as follows: 25µl of ATP releasate, 15µl of bead solution, 5µl of 20x ATP regenerating mix and 5µl of 300mM MBCD solution (prepared in LB buffer). The rest of the procedure was same as above.

15) Correlation analysis for colocalization of dynein and flotillin on the phagosome membrane.
A correlation analysis (shown in Fig S3-B and S3-D) was done using the pixel intensity patterns in Fig S3-A and S3-C respectively. The Labview 2013 (National Instruments) routine for calculation of 1D Cross Correlation was used to develop a program for this analysis. Two arrays of numbers, each corresponding to full rotation of 360 degrees along phagosome circumference, were given as input to this program :-

I. Dynein intensity **D** along phagosome circumference (e.g. Green line in Fig S3-A).
II. Flotillin intensity **F** along phagosome circumference (e.g. Red line in Fig S3-A).

The correlation coefficient (*R*) of intensity patterns D and F was calculated using the formula

$$R_J = \sum_{K=0}^{N-1} (D_K * F_{J+K})$$

For correlation index $J = -(N-1), -(N-2), \dots, -1, 0, 1, \dots, (N-2), (N-1)$

R was calculated using arrays D and F (Cross correlation; e.g. Black line in Fig S3-B). *R* was then calculated with both arrays taken as D (Dynein intensity autocorrelation; Green line in Fig S3-B). Lastly, *R* was calculated with both arrays taken as F (Flotillin intensity autocorrelation; Red line in Fig S3-B). Each correlation coefficient array was normalized by the maximum value in that array.

SUPPLEMENTAL REFERENCES

Barak, P., Rai, A., Dubey, A.K., Rai, P., and Mallik, R. (2014). Reconstitution of microtubule-dependent organelle transport. Methods Enzymol *540*, 231-248.

Barak, P., Rai, A., Rai, P., and Mallik, R. (2013). Quantitative optical trapping on single organelles in cell extract. Nat Methods *10*, 68-70.

Bligh, E.G., and Dyer, W.J. (1959). A rapid method of total lipid extraction and purification. Can J Biochem Physiol *37*, 911-917.

Blocker, A., Severin, F.F., Burkhardt, J.K., Bingham, J.B., Yu, H., Olivo, J.C., Schroer, T.A., Hyman, A.A., and Griffiths, G. (1997). Molecular requirements for bi-directional movement of phagosomes along microtubules. J Cell Biol *137*, 113-129.

Blocker, A., Severin, F.F., Habermann, A., Hyman, A.A., Griffiths, G., and Burkhardt, J.K. (1996). Microtubule-associated protein-dependent binding of phagosomes to microtubules. J Biol Chem *271*, 3803-3811.

Carter B.C., Shubeita G.T., and S.P., G. (2005). Tracking single particles: a user-friendly quantitative evaluation. Physical Biology *2*, 60-72.

Dermine, J.F., Goyette, G., Houde, M., Turco, S.J., and Desjardins, M. (2005). Leishmania donovani lipophosphoglycan disrupts phagosome microdomains in J774 macrophages. Cell Microbiol *7*, 1263-1270.

Desjardins, M., Huber, L.A., Parton, R.G., and Griffiths, G. (1994). Biogenesis of phagolysosomes proceeds through a sequential series of interactions with the endocytic apparatus. J Cell Biol *124*, 677-688.

Downie, M.M., and Kealey, T. (1998). Lipogenesis in the human sebaceous gland: glycogen and glycerophosphate are substrates for the synthesis of sebum lipids. J Invest Dermatol *111*, 199-205.

Fukui, Y., Yumura, S., and Yumura, T.K. (1987). Agar-overlay immunofluorescence: high-resolution studies of cytoskeletal components and their changes during chemotaxis. Methods Cell Biol *28*, 347-356.

Gotthardt, D., Warnatz, H.J., Henschel, O., Bruckert, F., Schleicher, M., and Soldati, T. (2002). High-resolution dissection of phagosome maturation reveals distinct membrane trafficking phases. Mol Biol Cell *13*, 3508-3520.

Goyette, G., Boulais, J., Carruthers, N.J., Landry, C.R., Jutras, I., Duclos, S., Dermine, J.F., Michnick, S.W., LaBoissiere, S., Lajoie, G.*, et al.* (2012). Proteomic characterization of phagosomal membrane microdomains during phagolysosome biogenesis and evolution. Mol Cell Proteomics *11*, 1365-1377.

Li, Y., Xu, S., Mihaylova, M.M., Zheng, B., Hou, X., Jiang, B., Park, O., Luo, Z., Lefai, E., Shyy, J.Y.*, et al.* (2011). AMPK phosphorylates and inhibits SREBP activity to attenuate hepatic steatosis and atherosclerosis in diet-induced insulin-resistant mice. Cell Metab *13*, 376-388.

Mallik R., Carter, B.C., Lex, S.A., King, S.J., and Gross, S.P. (2004). Cytoplasmic dynein functions as a gear in response to load. Nature *427*, 649-652.

Maxfield, F.R., and Wustner, D. (2012). Analysis of cholesterol trafficking with fluorescent probes. Methods Cell Biol *108*, 367-393.

Pollock N., Koonce, M.P., de Hostos, E.L., and Vale, R.D. (1998). In vitro microtubule-based organelle transport in wild-type Dictyostelium and cells overexpressing a truncated dynein heavy chain. Cell Motil Cytoskeleton *40*, 304-314.

Rai, A.K., Rai, A., Ramaiya, A.J., Jha, R., and Mallik, R. (2013). Molecular adaptations allow dynein to generate large collective forces inside cells. Cell *152*, 172-182.

Schroeder, F. (1982). Phagosomal membrane lipids of LM fibroblasts. J Membr Biol *68*, 141-150.

Soppina, V., Rai, A., and Mallik, R. (2009a). Simple non-fluorescent polarity labeling of Microtubules for Molecular Motor assays. Biotechniques *46*, 297-303.

Soppina, V., Rai, A.K., Ramaiya, A.J., Barak, P., and Mallik, R. (2009b). Tug-of-war between dissimilar teams of microtubule motors regulates transport and fission of endosomes. Proc Natl Acad Sci U S A *106*, 19381-19386.

Turco, S.J., Hull, S.R., Orlandi, P.A., Jr., Shepherd, S.D., Homans, S.W., Dwek, R.A., and Rademacher, T.W. (1987). Structure of the major carbohydrate fragment of the Leishmania donovani lipophosphoglycan. Biochemistry *26*, 6233-6238.
