# Supplementary figures and images for: Dynein Clusters into Lipid Microdomains on Phagosomes to Drive Rapid Transport toward Lysosomes
Source: Cell. 2016 Feb 11;164(4):722–34. doi: 10.1016/j.cell.2015.12.054 (PMC4752818; doi:10.1016/j.cell.2015.12.054)

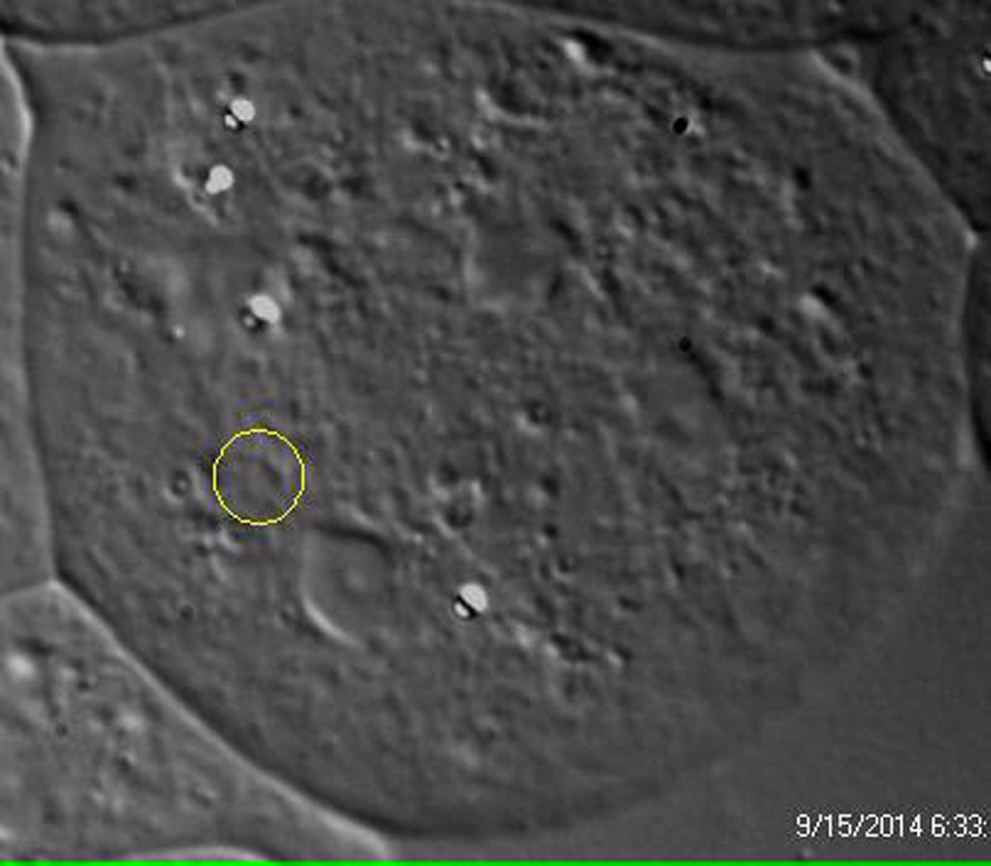

Supplement: Movie S1. Motion of an Early Phagosome Inside an Agar-Flattened Dictyostelium Cell, Related to Figure 1 — Movie runs in real time. Initial position of a motile early phagosome (EP) is circled. Various other organelles can also be seen moving vigorously inside the cell. [file mmc2.jpg]

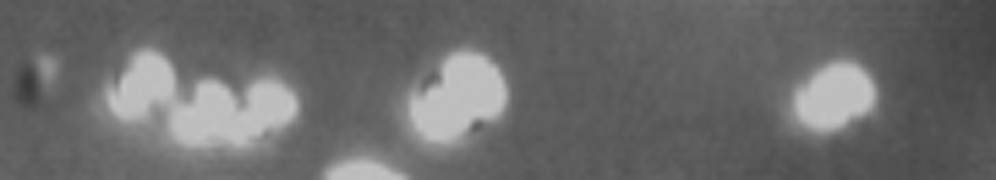

Supplement: Movie S2. In Vitro Motion of an Early Phagosome Purified from Dictyostelium along a Polarity-Labeled Microtubule, Related to Figure 1 — Movie runs in real time. The microtubule is not visible because it out of focus and has low contrast. magnetic beads labeling minus end of microtubule are indicated. The early phagosome (EP) moves bidirectionally (in back-and-forth manner; indicated by double headed red arrow) on the MT. An EP that is stuck on the MT is also shown. The EP is 759nm in diameter. [file mmc3.jpg]

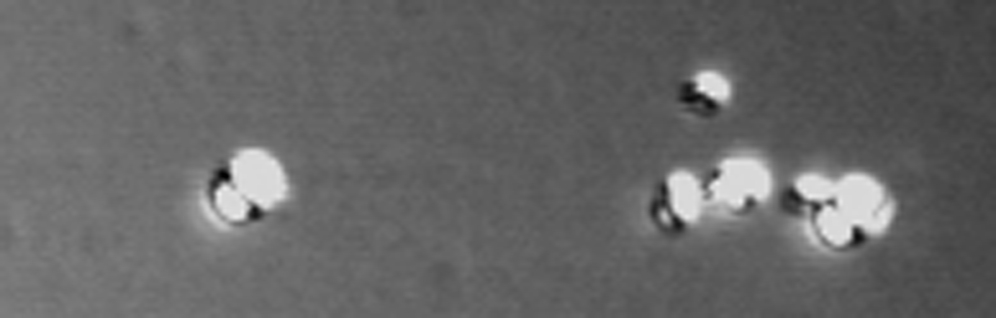

Supplement: Movie S3. In Vitro Motion of a Late Phagosome Purified from Dictyostelium along a Polarity-Labeled Microtubule, Related to Figure 1 — Movie runs in real time. The microtubule is not visible because it out of focus and has low contrast. Magnetic beads labeling minus end of microtubule are indicated. The late phagosome (LP) moves unidirectionally towards the minus end of the MT (indicated by red arrow). Another LP that had been placed on the same MT has moved to reached the extreme minus end of MT and is now stuck there. The LP is 759nm in diameter. [file mmc4.jpg]
